# Supplementary figures and images for: Combining acoustic survey and citizen science data yields enhanced species distribution models for tropical rainforest birds
Source: PLoS One. 2025 Jul 8;20(7):e0327944. doi: 10.1371/journal.pone.0327944 (PMC12237072; doi:10.1371/journal.pone.0327944)

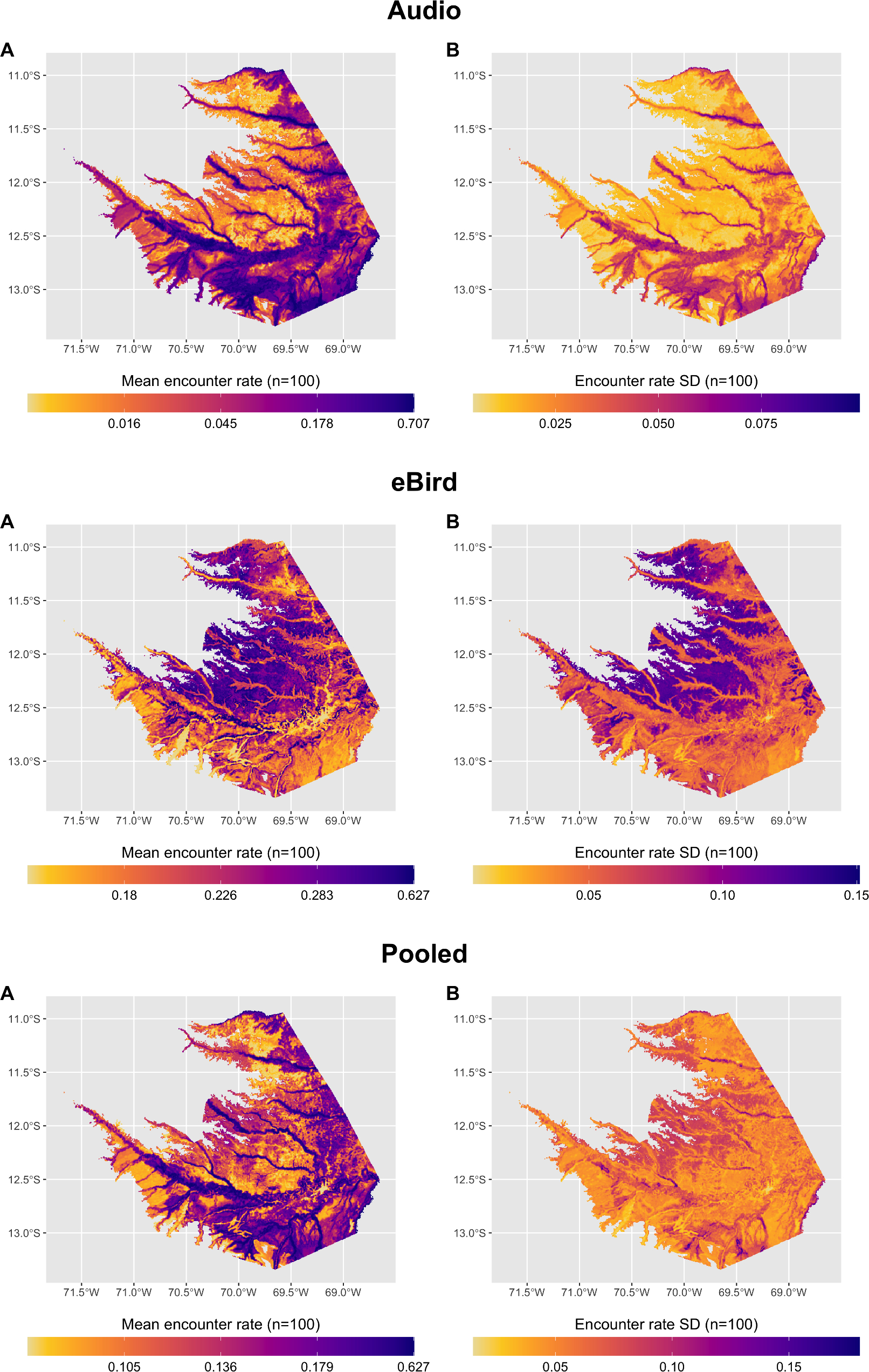

Supplement: S1 Fig — F. analis is a floodplain species. Pooled model offered high prediction accuracy both in natural and degraded habitats. (TIF) [file pone.0327944.s001.tif]

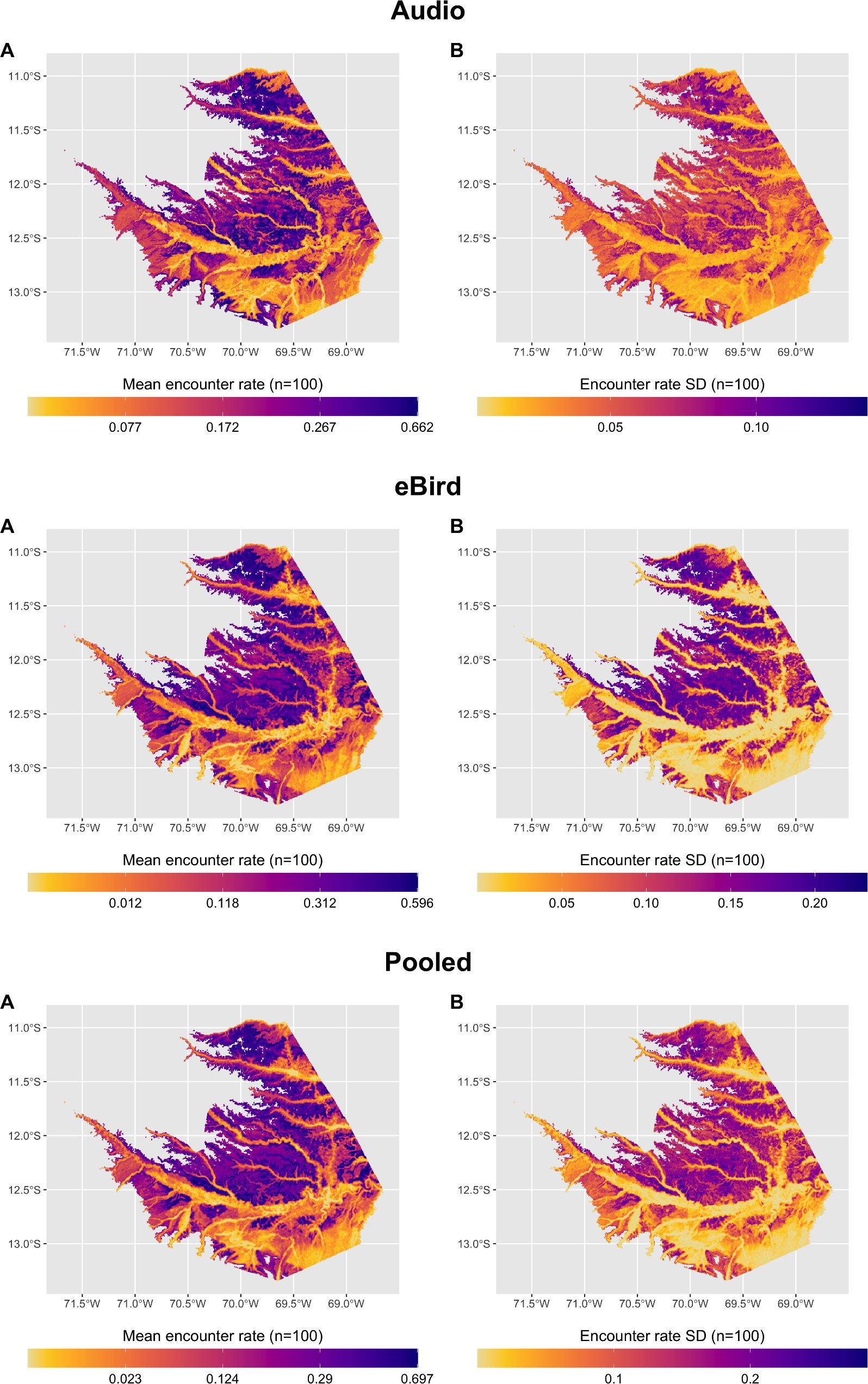

Supplement: S2 Fig — F. colma is a terra firme species. Pooled model offered high prediction accuracy both in natural and degraded habitats. (TIF) [file pone.0327944.s002.tif]

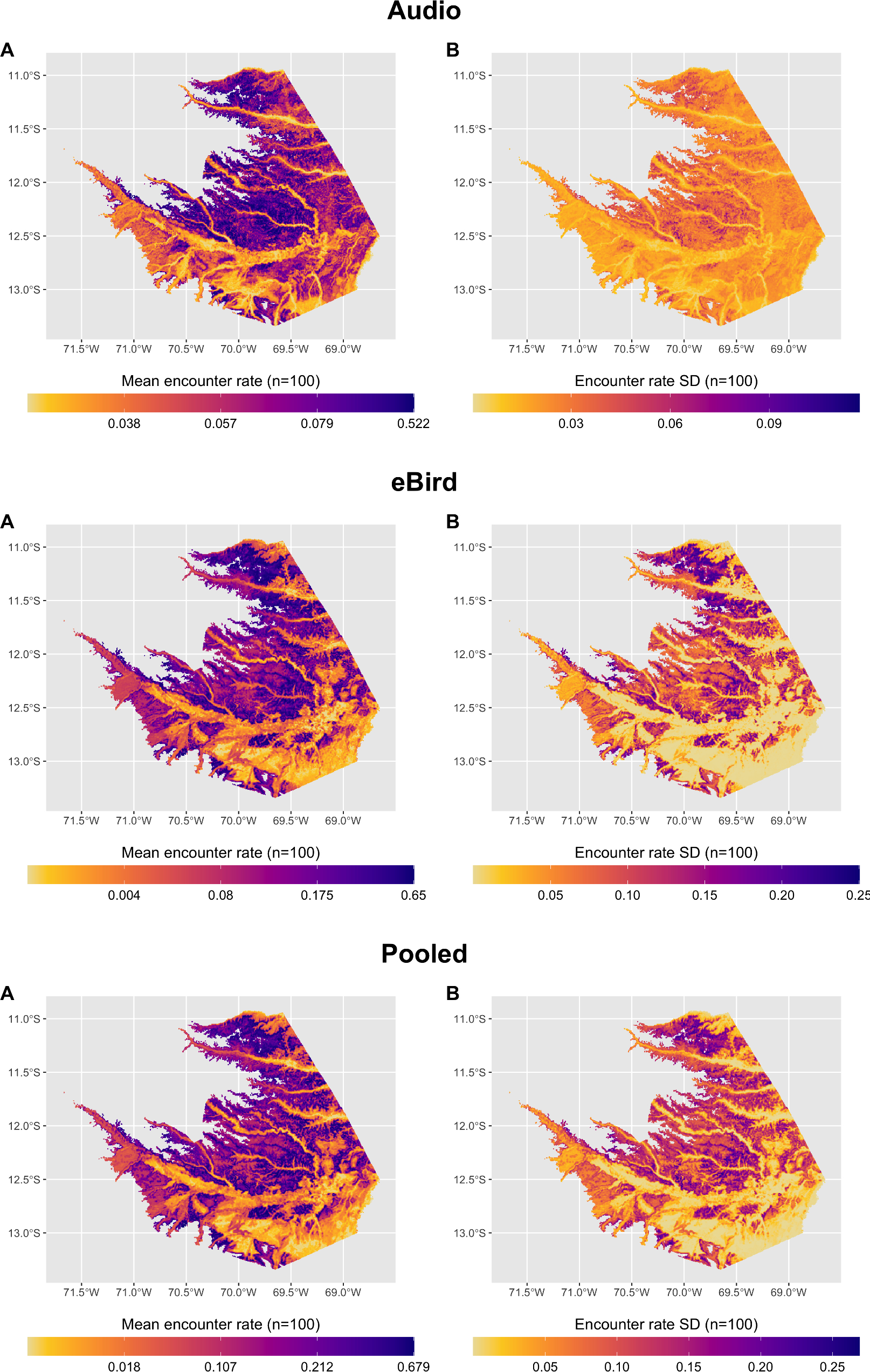

Supplement: S3 Fig — M. campanisona is a terra firme species. Pooled model offered high prediction accuracy both in natural and degraded habitats. (TIF) [file pone.0327944.s003.tif]

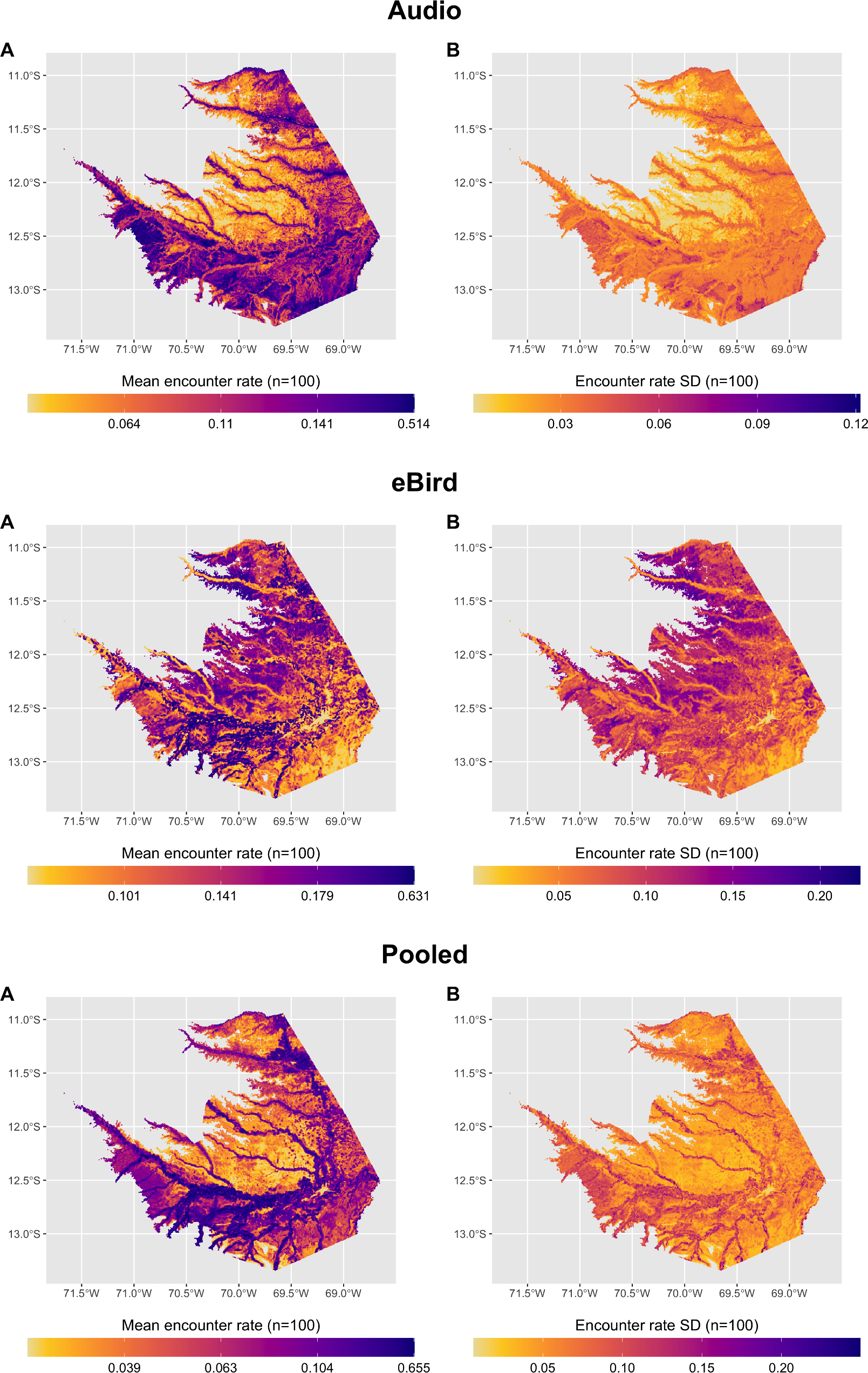

Supplement: S4 Fig — A. goeldii is a floodplain species. Pooled model offered high prediction accuracy both in natural and degraded habitats. (TIF) [file pone.0327944.s004.tif]

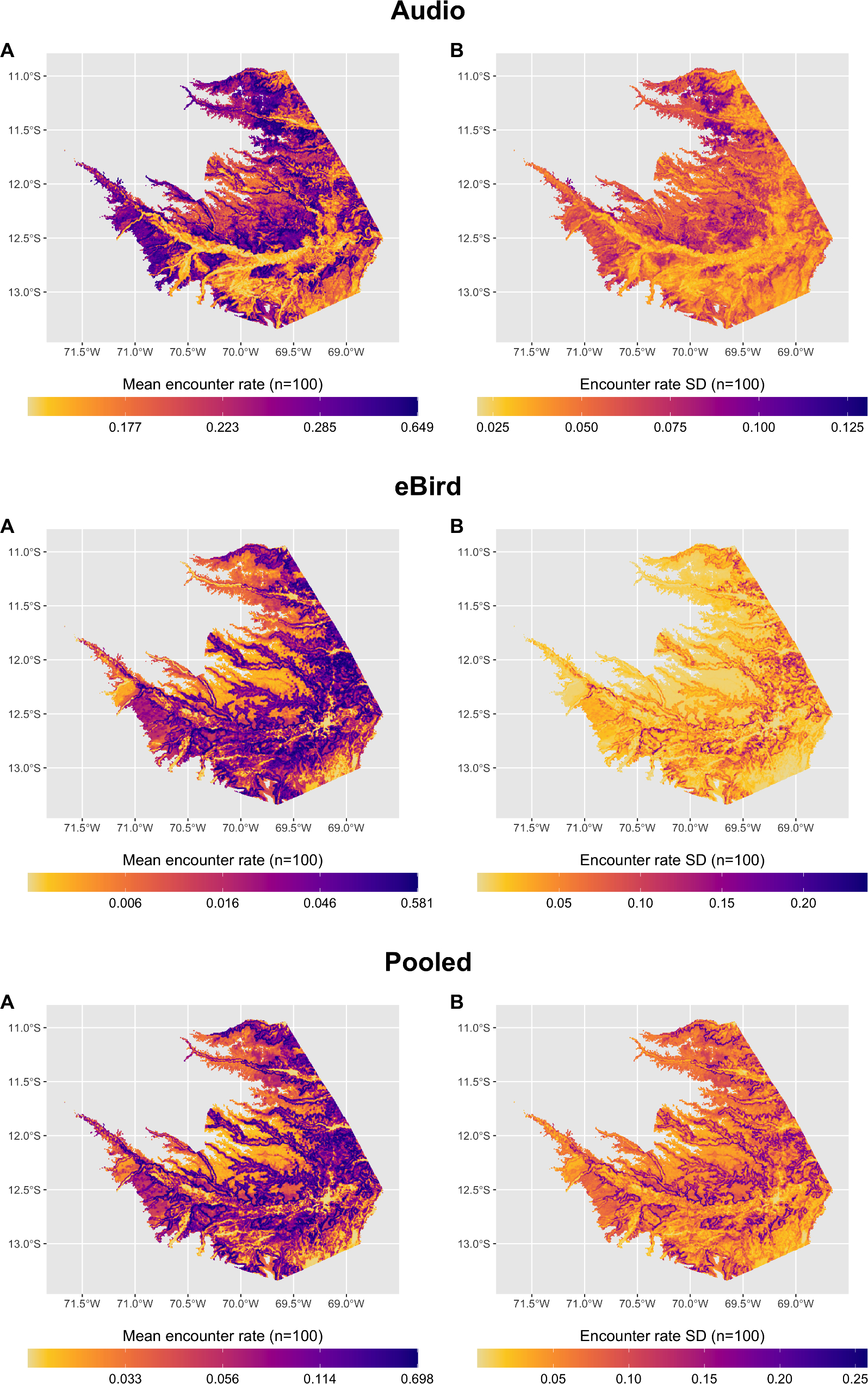

Supplement: S5 Fig — O. salvini is a terra firme species. Pooled model offered high prediction accuracy both in natural and degraded habitats. (TIF) [file pone.0327944.s005.tif]

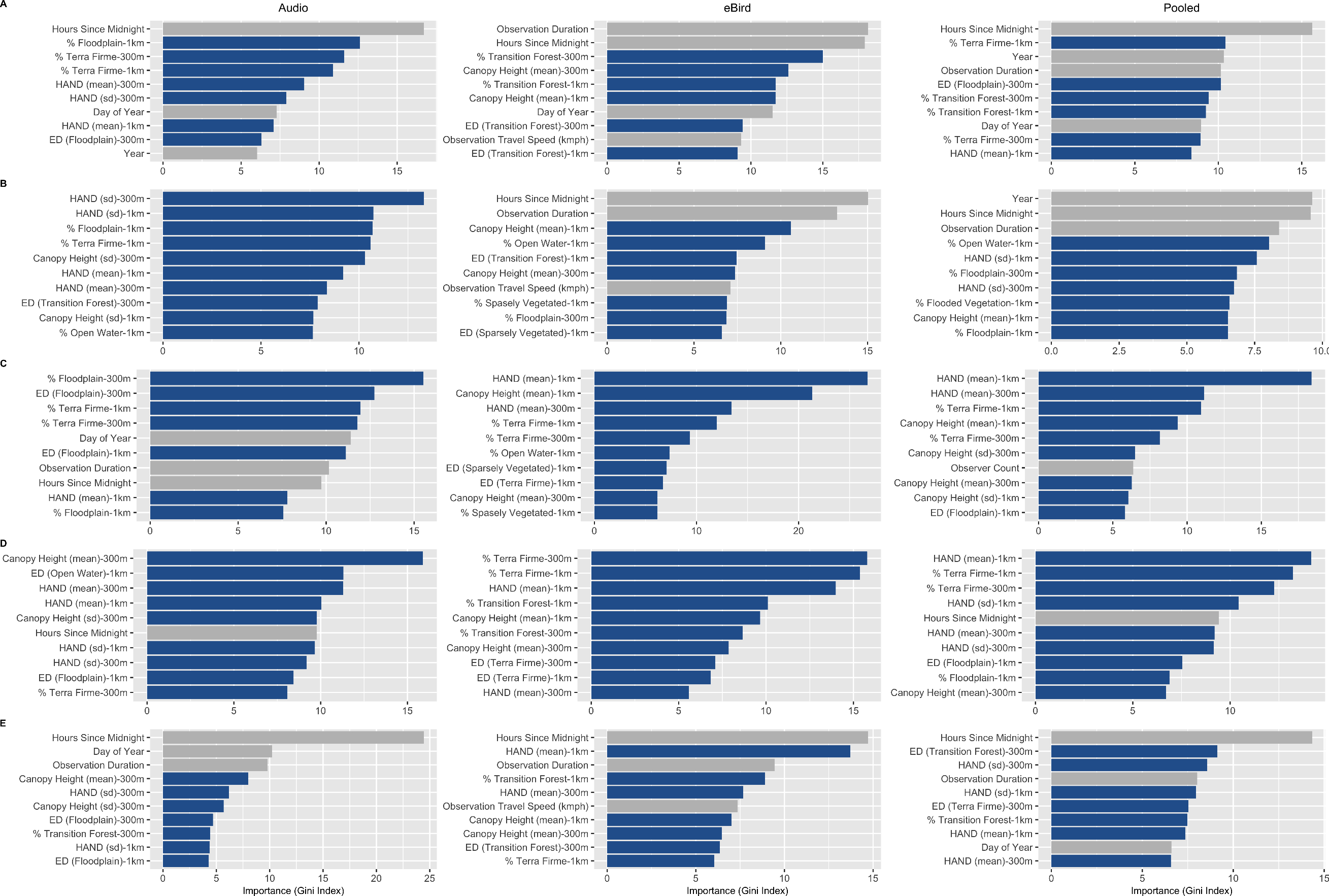

Supplement: S6 Fig — A = F. analis, B = A. goeldii, C = F. colma, D = M. campanisona, E = O. salvini. Environmental factors are indicated in blue; detection covariates are indicated in gray. Pooled models generally did a good job of assigning high rankings to habitat variables thought to be important to species occurrence, though relative ranks varied between species. (TIF) [file pone.0327944.s006.tif]

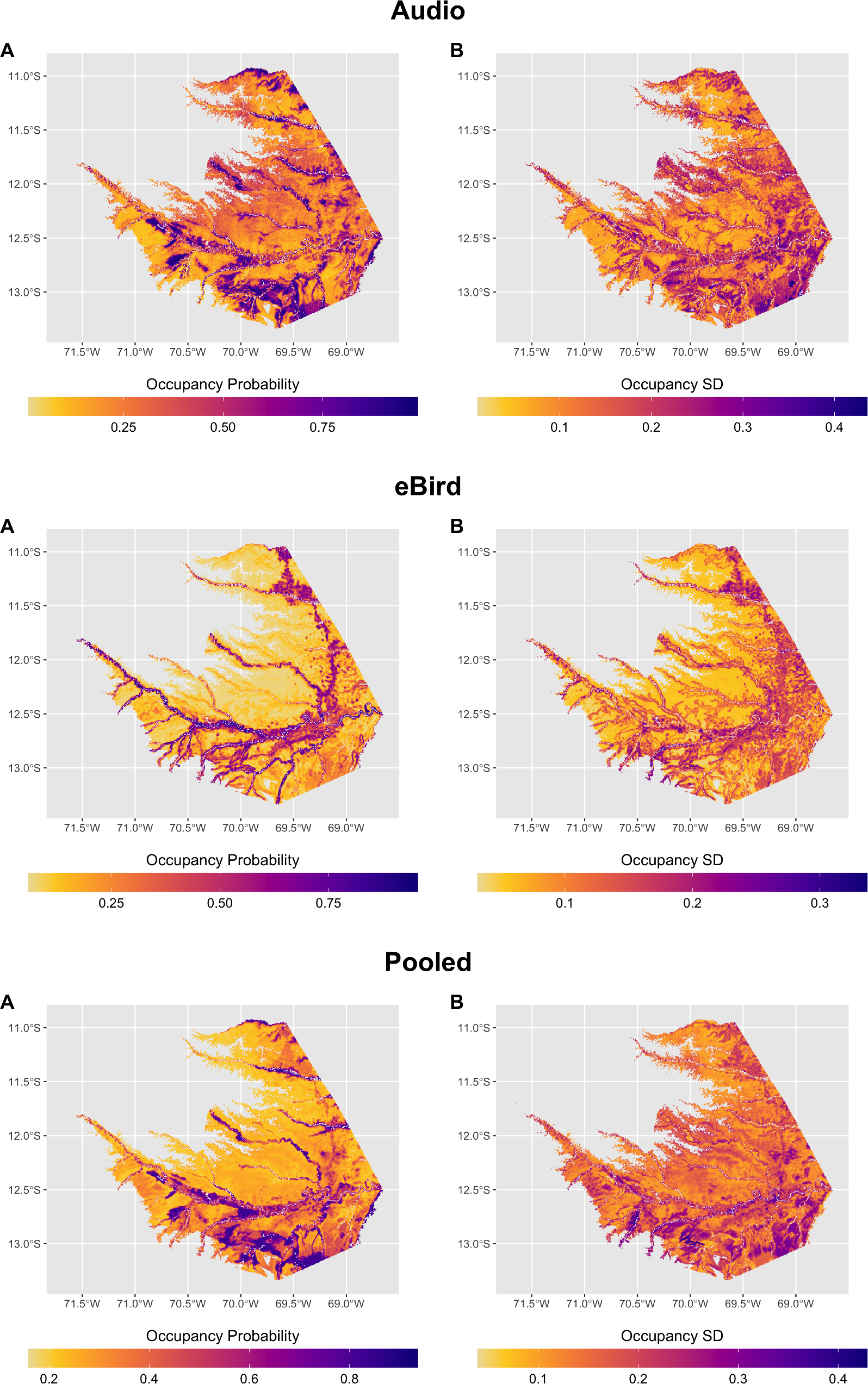

Supplement: S7 Fig — F. analis is a floodplain species. Pooled model offered high prediction accuracy both in natural and degraded habitats. (TIF) [file pone.0327944.s007.tif]

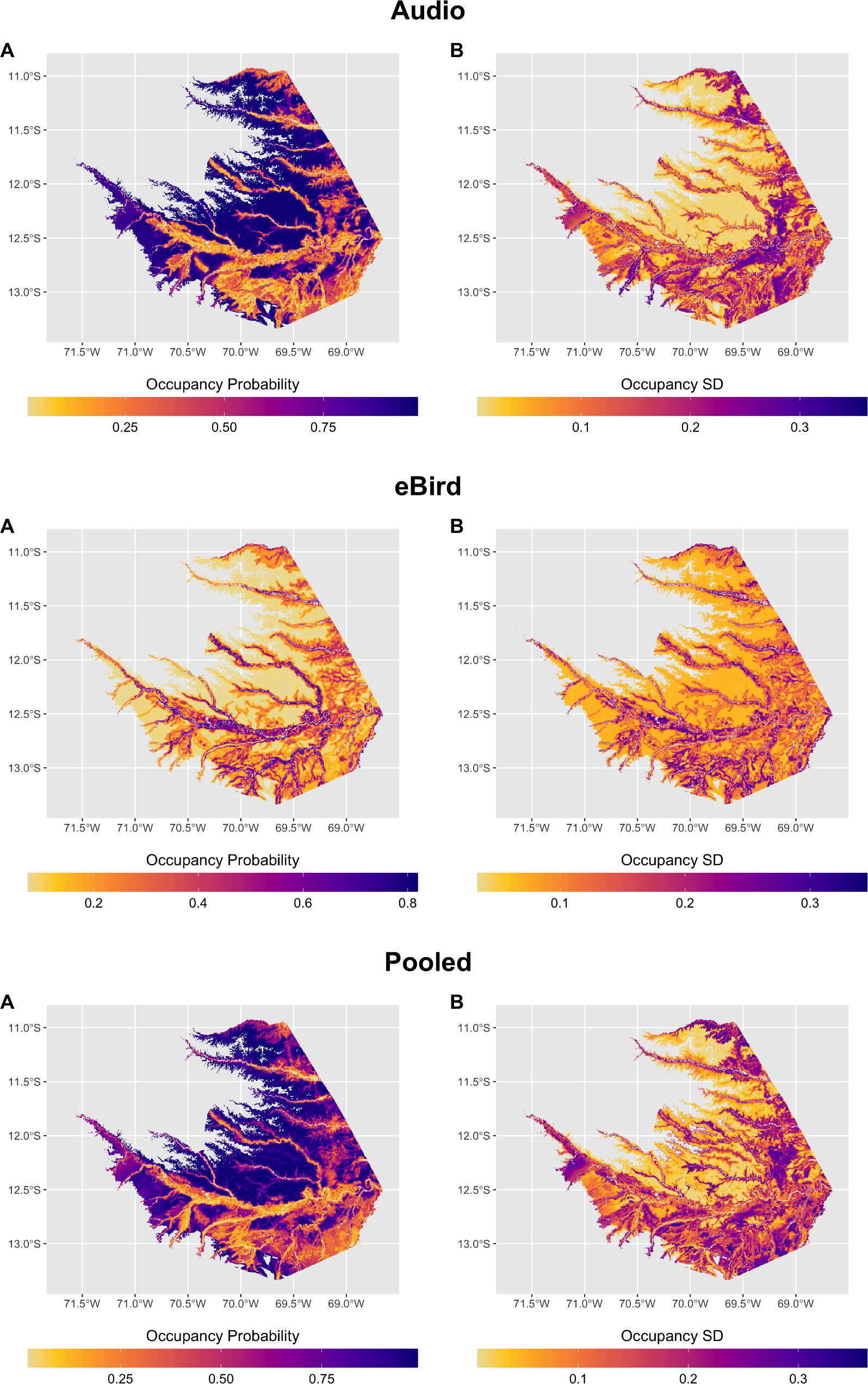

Supplement: S8 Fig — F. colma is a terra firme species. Pooled model offered high prediction accuracy both in natural and degraded habitats. (TIF) [file pone.0327944.s008.tif]

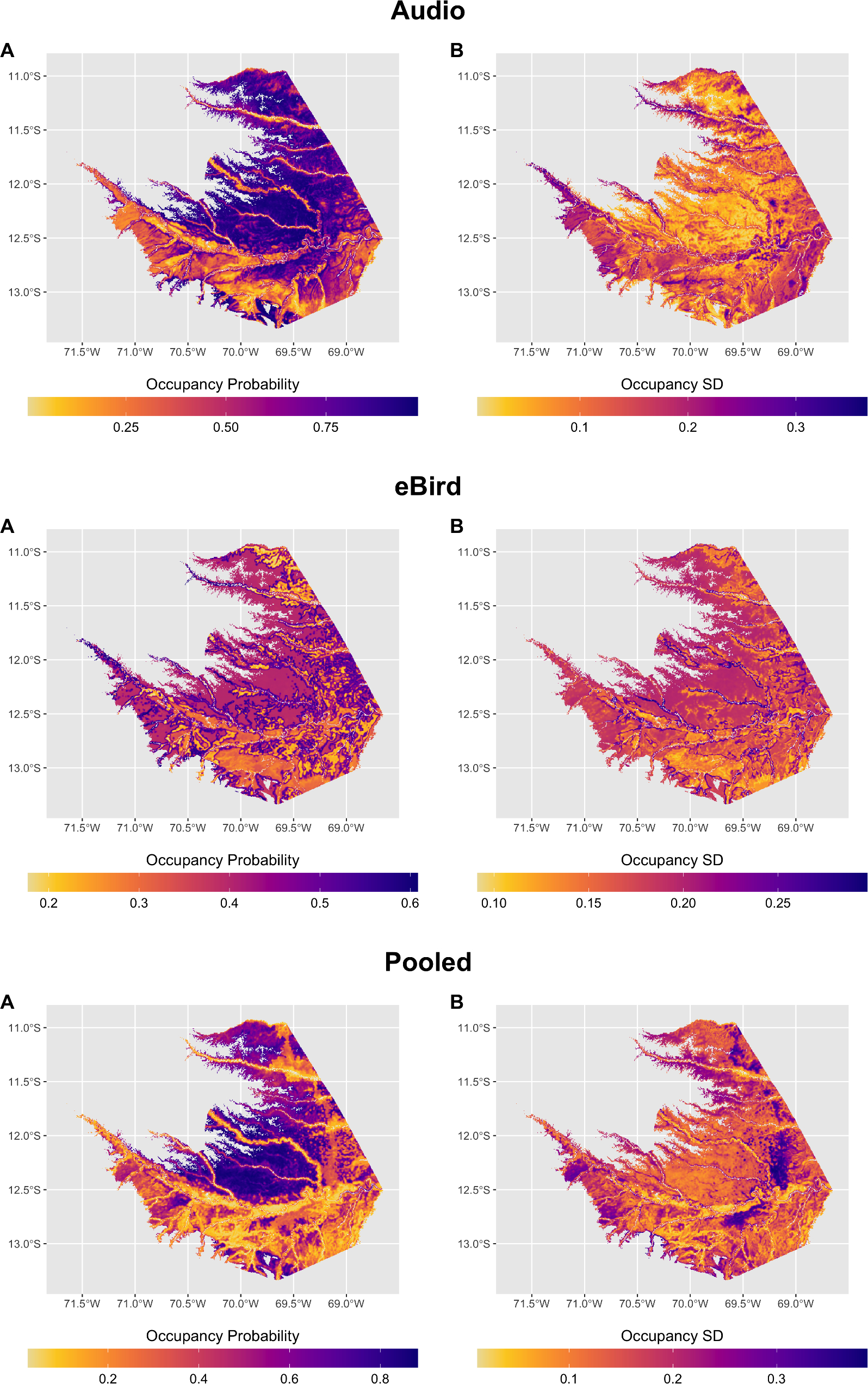

Supplement: S9 Fig — M. campanisona is a terra firme species. Pooled model offered high prediction accuracy both in natural and degraded habitats. (TIF) [file pone.0327944.s009.tif]

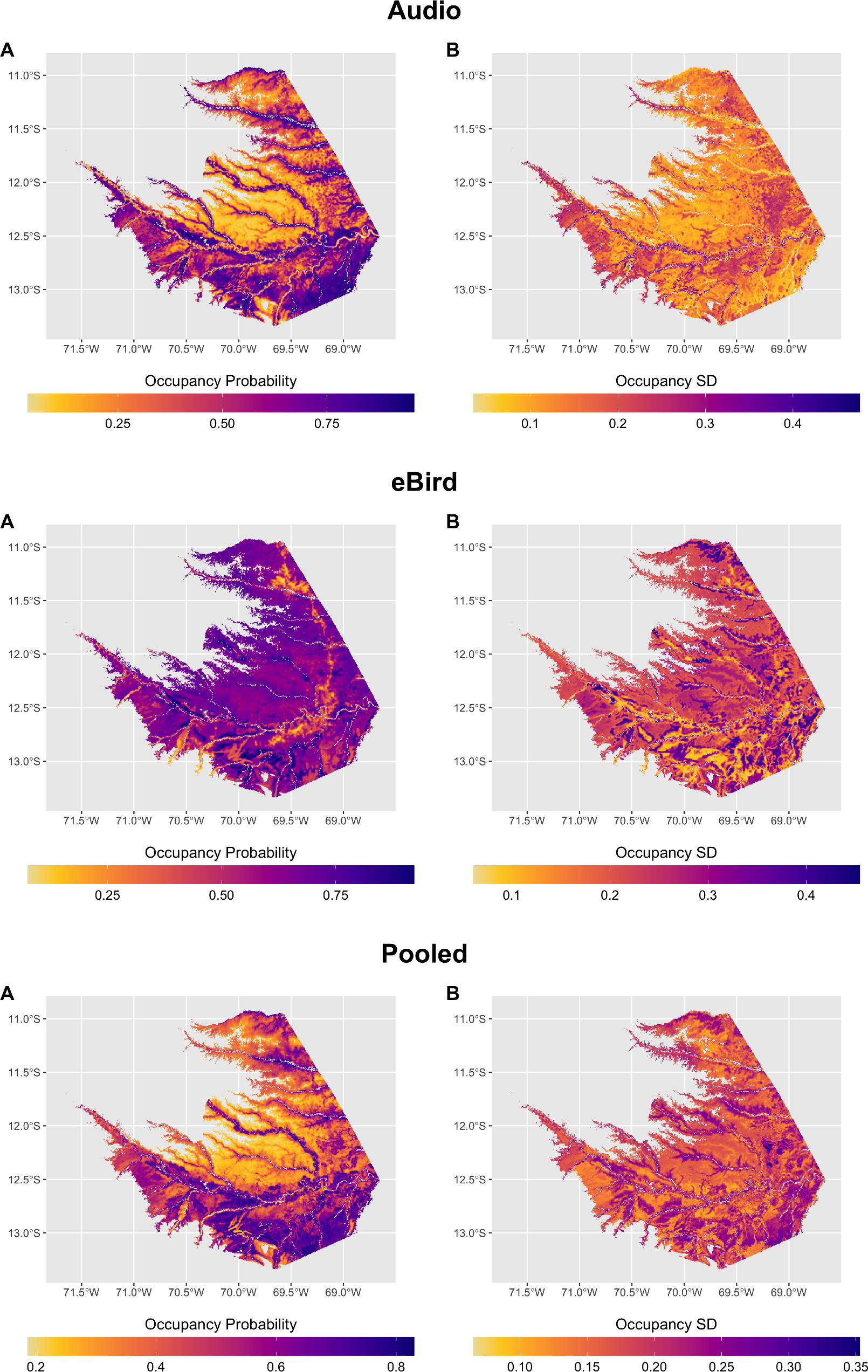

Supplement: S10 Fig — A. goeldii is a floodplain species. Pooled model offered high prediction accuracy both in natural and degraded habitats. (TIF) [file pone.0327944.s010.tif]

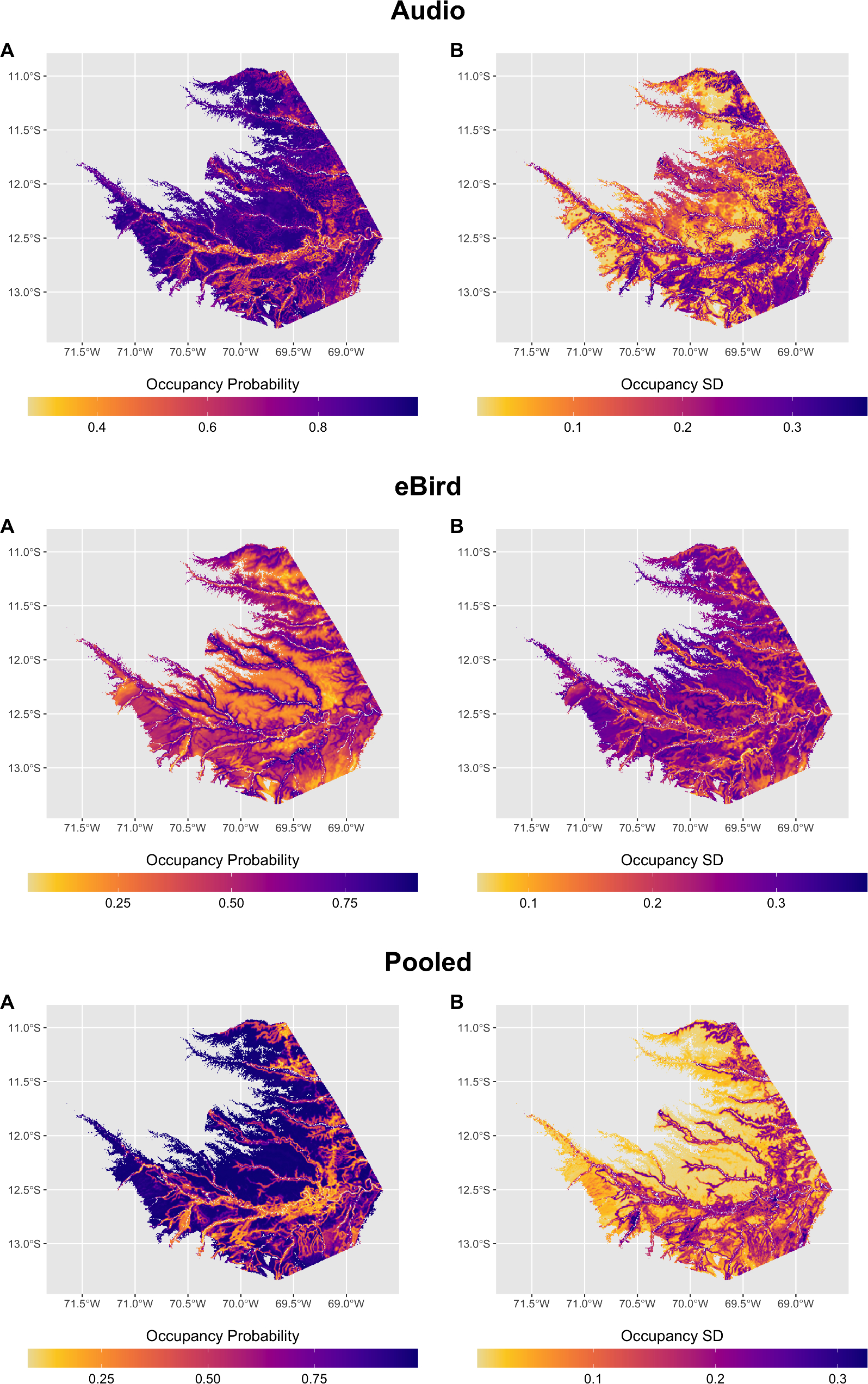

Supplement: S11 Fig — O. salvini is a terra firme species. Pooled model offered high prediction accuracy both in natural and degraded habitats. (TIF) [file pone.0327944.s011.tif]

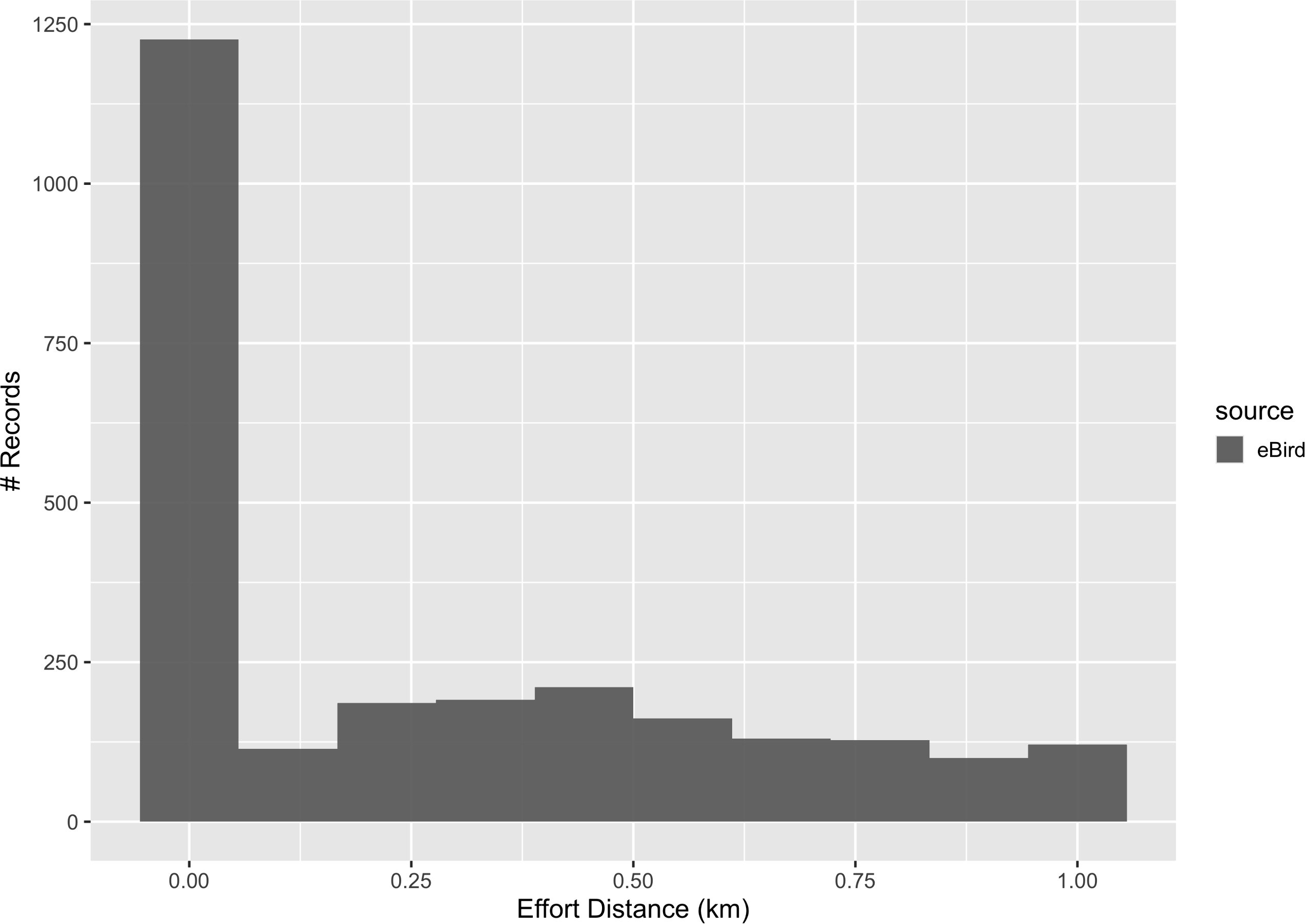

Supplement: S12 Fig — Using eBird data for inference across the short, steep ecological gradients in the Neotropics is challenging, especially when including travelling checklists, as the coordinate point assigned to checklists often does not accurately capture the areas covered by observers. Constraining eBird data to checklists that cover distances of >1 km distance reduces, but does not eliminate this bias, and while more restrictive cutoffs would further reduce bias, doing so generally also yields dramatic reductions in sample size. (TIF) [file pone.0327944.s012.tif]

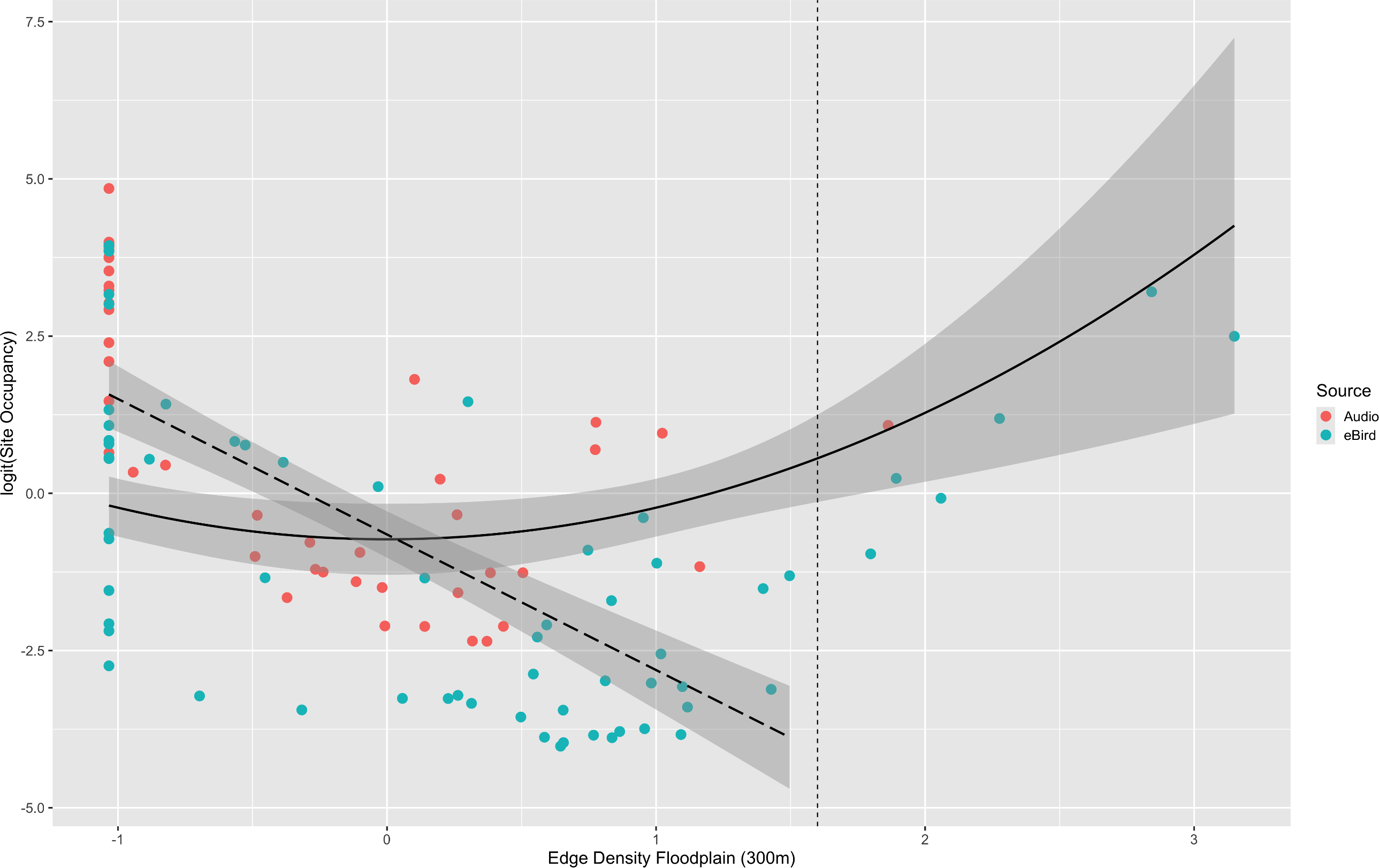

Supplement: S13 Fig — Solid line indicates the shape of the relationship predicted by the highest performing model bootstrap. The quadratic shape of this relationship is mainly driven by a cluster of eBird site-visits with edge density values >1.6 (vertical dotted line) that have high predicted site occurrence values; all of these are checklists from popular birding guides at large, popular ecolodges. Removing these points leads to a relationship that is more ecologically plausible (dotted line). (TIF) [file pone.0327944.s013.tif]

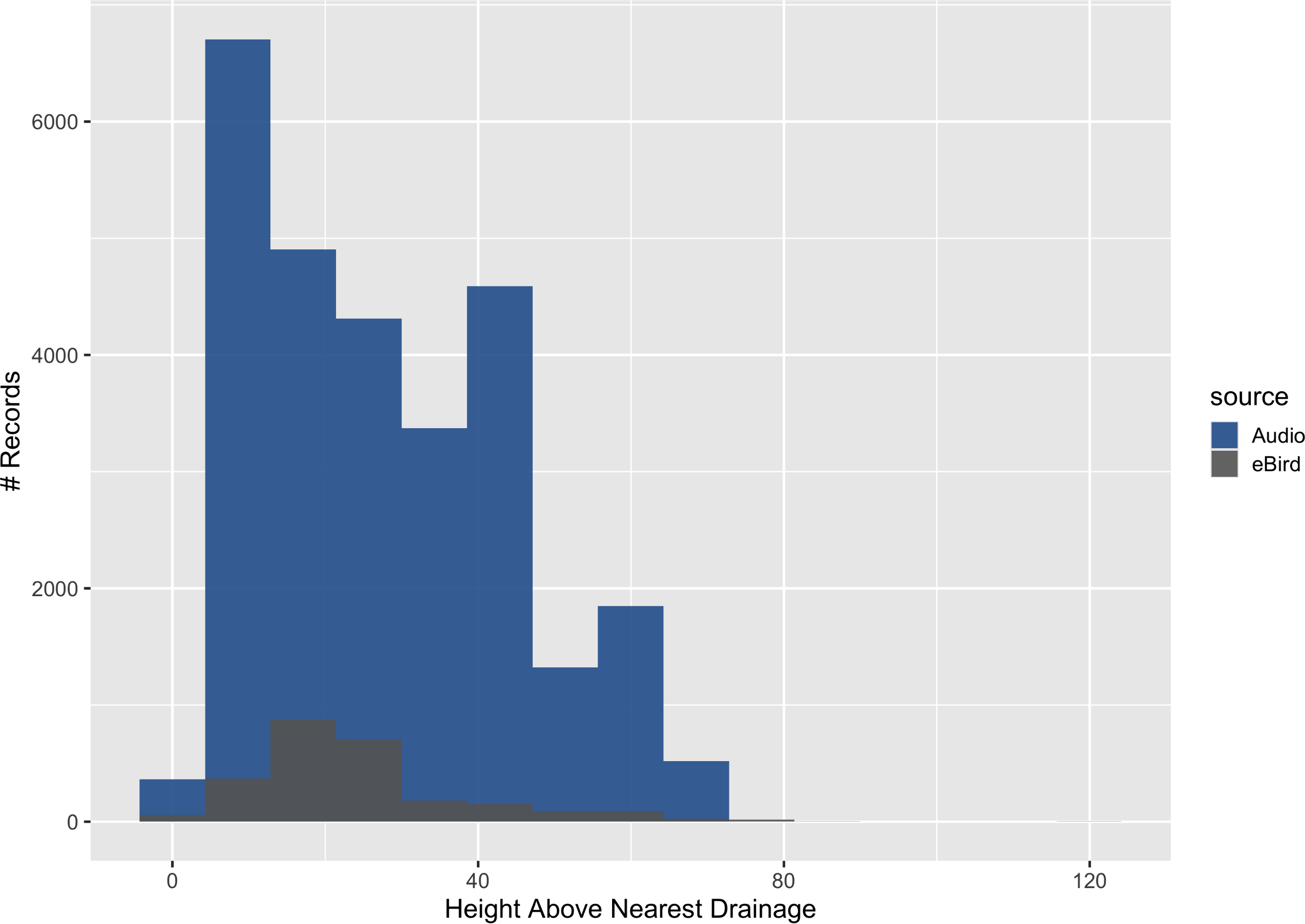

Supplement: S14 Fig — Many eBird hotspots are near the major rivers that serve as the main means of transportation in the lowland Amazon, causing the overall distribution of HAND within the eBird data fraction to be biased toward the lower end of the critical floodplain-terra firme ecological gradient. (TIF) [file pone.0327944.s014.tif]
