# Supplementary material for: Combining acoustic survey and citizen science data yields enhanced species distribution models for tropical rainforest birds
Source: PLoS One. 2025 Jul 8;20(7):e0327944. doi: 10.1371/journal.pone.0327944 (PMC12237072; doi:10.1371/journal.pone.0327944)
Supplement: S1 Table — All classification was performed using BirdNET v2.4. (DOCX) [file pone.0327944.s015.docx]

| **Parameter** | **Argument name** | **Value** |
| --- | --- | --- |
| **Latitude** | *--lat* | -12.5692243 |
| **Longitude** | *--lon* | -70.1002836 |
| **Week** | *--week* | -1 |
| **Sensitivity** | *--sensitivity* | 1 |
| **Minimum confidence threshold** | *--min_conf* | 0.1 |
| **Overlap** | *--overlap* | 2.75 |
