# Supplementary material for: Combining acoustic survey and citizen science data yields enhanced species distribution models for tropical rainforest birds
Source: PLoS One. 2025 Jul 8;20(7):e0327944. doi: 10.1371/journal.pone.0327944 (PMC12237072; doi:10.1371/journal.pone.0327944)
Supplement: S1 File — Additional details on the manner in which acoustic monitoring data was collected. (DOCX) [file pone.0327944.s019.docx]

**S1 File**

Acoustic survey data for this project were collected over the course of 3 seasons spanning 5 years. During all field seasons, recorders were placed ~1 m above the ground with the microphone facing towards the ground, which is optimal for recording understory (most activity <= 2m above ground) and terrestrial birds. Additionally, all surveys across all seasons were recorded in mono as we do not have any specific use for stereo audio at this time. However, the deployment strategies used in each season changed based on equipment availability and project goals, which we summarize below.

Survey data from 2019 and 2020 were collected as part of a pilot study and subsequent full monitoring project for tinamous (Tinamidae), respectively. During this set of surveys, 10 Cornell Lab of Ornithology Swift autonomous recording units (ARUs) were cycled across 34 survey points covering the terra firme-floodplain gradient (Fig 1) from late July to early October 2019 (dry season in southeastern Peru) and then again at the same sites from early January to late February 2020 (wet season). Recording durations were 2-23 days per site during the pilot study and 21 days per site during the wet season. As these surveys were conducted at a time when very limited infrastructure was available to support acoustic monitoring at this field station, deployment strategies prioritized storage space and battery life efficiency. Recorder sampling frequency was set to 16 kHz to remove acoustic frequencies greater than 8 kHz; this allowed us to reduce recording file size and extend battery life while retaining acoustic fidelity in the frequency bands in which our target species’ vocalizations occur (<3.5 kHz). Microphone gain was left at the default -33 dB. Although this survey methodology was designed for tinamous, these parameters are also appropriate for capturing the vocalizations of our target species as the two clades broadly overlap in forest strata and acoustic frequency bands. In addition, we chose to mainly record the dawn and dusk choruses when our study species are most vocal: in the 2019 pilot study, recorders were active 5:00 and 7:30 hrs and 16:00 and 18:30 hrs; in 2020, recording was conducted 3:30 to 7:30 hrs and from 14:30 to 18:30 hrs, as well as 15 minutes every half-hour between 8:30 hrs and 14:00 hrs and between 19:30 and 24:00 hrs. Survey audio was captured as a series of WAV files of 30 min duration (except for files outside the dawn and dusk chorus which were 15 minutes long).

The remainder of the acoustic survey data was collected from mid-May to mid-September 2024. Recording was conducted at 19 recording sites at new sites along the EBLA trail system that were chosen so that they provided better spatial resolution within the ecotone between terra firme and floodplain (Fig 1). From May to mid-August, 4 Frontier Labs Bar-LT recorders were deployed for 3 30-day long deployments at 4 sites. Starting in mid-August and continuing through the end of the season, 6 Cornell Lab of Ornithology SwiftOne ARUs and 9 Frontier Labs BAR-LT recorders were deployed for an additional single 21-day long deployment at the remaining 15 sites. As there was greater support infrastructure available for acoustic monitoring at this time, which included having access to much larger SD cards, larger hard drives for in-the-field data archiving, and higher capacity rechargeable batteries, we were able to expand the data collection scope to a 24 hour continuous recording strategy and 44.1 kHz (May-Aug) or 48 kHz (Aug-Sep) sampling frequencies. WAV file durations were 30 minutes during May-August and 1 hr from August-September. Microphone gain was set to -40 dB in order to match other acoustic monitoring projects being conducted by collaborators in Madre de Dios and Cusco departments during this time period.

As a final note, we think it important to reiterate that working with data from multiple recording seasons, especially with differing deployment strategies, requires careful consideration to ensure that inferences from different seasons are comparable to one another. We feel we have satisfied these concerns by (1) thresholding the 2024 audio separately from the 2019-2020 audio to account for differences in microphone quality, digital sampling rate, recorder gain, and possible changes in the environment between years, (2) filtering the continuous survey audio from 2024 so that we only use audio from the same times of day that the recorders were active during the 2020 surveys, and (3) including year as a covariate in all of our statistical models to account for residual error due to deployment strategy remaining after the first two steps. However, we feel there are multiple correct ways to approach this issue and that the most optimal strategy for a given project is likely highly context specific. For example, as all of our study species have sub-8 kHz vocalizations, the more limited frequency bandwidth available to us in the 2019 and 2020 audio files is unlikely to have resulted in lower fidelity within the relevant frequency ranges relative to the 2024 audio. However, this would not have been the case if we wished to repeat this project for bird species whose vocalizations have >8 kHz components, and in this hypothetical scenario two possible strategies to avoid biasing the classifications towards the higher fidelity audio pool would have been to remove the pre-2024 audio outright or to insert an additional processing step to resample the 2024 audio down to 16 kHz prior to performing BirdNET classification.
