# Supplementary material for: Combining acoustic survey and citizen science data yields enhanced species distribution models for tropical rainforest birds
Source: PLoS One. 2025 Jul 8;20(7):e0327944. doi: 10.1371/journal.pone.0327944 (PMC12237072; doi:10.1371/journal.pone.0327944)
Supplement: S3 File — Additional details about the top audio-only, eBird-only, and pooled occupancy model bootstraps from each species estimates, 95% BCIs, R-hat values, and model AUCs. (DOCX) [file pone.0327944.s021.docx]

**1. Occupancy model parameter estimates, *Formicarius analis***

| **Model** | **Process** | **Model Covariates** | **Est.** | **Lower 95% BCI** | **Upper 95% BCI** | **Rhat** | **PR-AUC** |
| --- | --- | --- | --- | --- | --- | --- | --- |
| **Audio** | Occurrence (ψ) | (Intercept) | -0.449 | -2.106 | 1.286 | 1.000 | **0.926** |
|  |  | Canopy Height Mean (300m) | 1.249 | -0.164 | 2.723 | 1.000 |  |
|  |  | **Percent Cover Floodplain (1 km)** | **2.623** | **1.165** | **4.144** | **1.001** |  |
|  |  | Edge Density Open Water (300m)^2 | 0.695 | -1.932 | 3.302 | 1.001 |  |
|  |  | Percent Cover Open Water (300m)^2 | 1.516 | -0.391 | 3.534 | 1.001 |  |
|  |  | Edge Density Floodplain (300m)^2 | 1.398 | -0.094 | 3.103 | 1.000 |  |
|  |  | **Edge Density Terra Firme (300m)^2** | **-1.455** | **-3.005** | **-0.132** | **1.000** |  |
|  | Detection (p) | **(Intercept)** | **1.410** | **0.673** | **2.252** | 1.001 |  |
|  |  | **Hours of Day** | **-3.183** | **-4.614** | **-1.841** | 1.002 |  |
| **eBird** | Occurrence (ψ) | (Intercept) | 1.596 | -0.144 | 3.543 | 1.000 | **0.947** |
|  |  | Canopy Height SD (1 km) | 1.119 | -0.141 | 2.425 | 1.000 |  |
|  |  | **Canopy Height Mean (1 km)^2** | **-1.443** | **-2.854** | **-0.046** | **1.001** |  |
|  |  | Edge Density Open Water (1 km)^2 | -0.996 | -2.381 | 0.173 | 1.001 |  |
|  | Detection (p) | **(Intercept)** | **-0.917** | **-1.503** | **-0.334** | **1.000** |  |
|  |  | **Hours of Day** | **-0.901** | **-1.442** | **-0.385** | **1.000** |  |
|  |  | Effort Hours | 0.510 | -0.066 | 1.251 | 1.000 |  |
| **Pooled** | Occurrence (ψ) | (Intercept) | 0.187 | -1.067 | 1.459 | 1.000 | **0.905** |
|  |  | Canopy Height SD (300m) | 0.864 | -0.407 | 2.248 | 1.001 |  |
|  |  | Percent Cover Open Water (1 km) | 1.651 | -0.035 | 3.374 | 1.000 |  |
|  |  | Edge Density Open Water (1 km) | -1.183 | -2.679 | 0.282 | 1.000 |  |
|  |  | **Edge Density Floodplain (1 km)** | **1.338** | **0.073** | **2.636** | **1.000** |  |
|  |  | **Percent Cover Floodplain (1 km)^2** | **1.499** | **0.413** | **2.654** | **1.000** |  |
|  | Detection (p) - eBird | **(Intercept)** | **-1.758** | **-2.283** | **-1.276** | **1.000** |  |
|  |  | **Hours of Day** | **-0.649** | **-1.114** | **-0.163** | **1.001** |  |
|  |  | Effort Hours | -0.088 | -0.539 | 0.369 | 1.000 |  |
|  | Detection (p) - Audio | **(Intercept)** | **1.178** | **0.428** | **2.011** | **1.001** |  |
|  |  | **Hours of Day** | **-2.997** | **-4.560** | **-1.650** | **1.002** |  |

All predictor scores are logit-scaled and unitless. Bolded predictors are significant (95% BCI does not include zero). The best performing model for this species was the eBird-only model, which showed that site occupancy was associated with intermediate mean canopy height. The best audio-only and pooled models measured slightly worse, but revealed other associations not present in the eBird-only model, including with increasing percent cover of floodplain, intermediate levels of terra firme edge density, and increasing edge density of floodplain.

**2. Occupancy model parameter estimates, *Akeletos goeldii***

| **Model** | **Process** | **Model Covariates** | **Est.** | **Lower 95% BCI** | **Upper 95% BCI** | **Rhat** | **PR-AUC** |
| --- | --- | --- | --- | --- | --- | --- | --- |
| **Audio** | Occurrence (ψ) | (Intercept) | 0.949 | -0.805 | 2.805 | 1.000 | **0.807** |
|  |  | HAND Mean (1 km) | -1.057 | -2.467 | 0.131 | 1.000 |  |
|  |  | Canopy SD (1 km)^2 | -1.191 | -2.747 | 0.164 | 1.003 |  |
|  | Detection (p) | **(Intercept)** | **-1.955** | **-3.005** | **-0.911** | **1.002** |  |
|  |  | Hours of Day | -1.278 | -3.991 | 1.656 | 1.001 |  |
|  |  | Hours of Day^2 | 1.624 | -0.250 | 3.550 | 1.034 |  |
| **eBird** | Occurrence (ψ) | (Intercept) | -0.212 | -1.893 | 1.530 | 1.001 | **0.812** |
|  |  | **Canopy Height Mean (1 km)** | **1.839** | **0.036** | **3.775** | **1.001** |  |
|  |  | **Edge Density Transition Forest (1 km)** | **1.755** | **0.035** | **3.658** | **1.002** |  |
|  | Detection (p) | **(Intercept)** | **-1.988** | **-2.790** | **-1.203** | **1.000** |  |
|  |  | **Effort Hours** | **0.804** | **0.269** | **1.400** | **1.000** |  |
| **Pooled** | Occurrence (ψ) | (Intercept) | 0.663 | -0.831 | 2.654 | 1.002 | **0.727** |
|  |  | Percent Cover Floodplain (300m) | 0.804 | -0.246 | 2.198 | 1.000 |  |
|  | Detection (p) - eBird | **(Intercept)** | **-2.260** | **-3.096** | **-1.444** | **1.001** |  |
|  |  | **Effort Hours** | **0.746** | **0.245** | **1.288** | **1.001** |  |
|  | Detection (p) - Audio | **(Intercept)** | **-2.305** | **-3.366** | **-1.156** | **1.002** |  |
|  |  | Hours of Day | -1.395 | -4.201 | 1.412 | 1.000 |  |
|  |  | Hours of Day^2 | 1.624 | -0.300 | 3.751 | 1.008 |  |

All predictor scores are logit-scaled and unitless. Bolded predictors are significant (95% BCI does not include zero). The best-performing model for this species was the eBird-only model, which indicated that site occupancy was positively associated with canopy height and edge density of transition forest. The audio-only and pooled models performed less well, indicating tentative associations with decreasing HAND, intermediate values of canopy height SD, and increasing percent cover of floodplain.

**3. Occupancy model parameter estimates, *F. colma***

| **Model** | **Process** | **Model Covariates** | **Est.** | **Lower 95% BCI** | **Upper 95% BCI** | **Rhat** | **PR-AUC** |
| --- | --- | --- | --- | --- | --- | --- | --- |
| **Audio** | Occurrence (ψ) | (Intercept) | 0.667 | -0.841 | 2.109 | 1.001 | **0.939** |
|  |  | **HAND Mean (1 km)** | **2.195** | **0.217** | **4.082** | **1.001** |  |
|  |  | Edge Density Floodplain (300m) | -1.542 | -3.257 | 0.043 | 1.006 |  |
|  | Detection (p) | **(Intercept)** | **-1.813** | **-2.937** | **-0.695** | **1.002** |  |
|  |  | **Day of Year** | **-1.199** | **-1.777** | **-0.626** | **1.000** |  |
|  |  | Hours of Day | -2.037 | -4.601 | 0.493 | 1.002 |  |
|  |  | Hours of Day^2 | 1.728 | -0.450 | 3.921 | 1.003 |  |
| **eBird** | Occurrence (ψ) | (Intercept) | -1.221 | -3.739 | 1.710 | 1.000 | **0.592** |
|  |  | Edge Density Transition Forest (1 km)^2 | -1.576 | -4.152 | 0.964 | 1.004 |  |
|  | Detection (p) | (Intercept) | -1.260 | -3.825 | 1.518 | 1.001 |  |
|  |  | Day of Year^2 | -1.383 | -4.411 | 1.694 | 1.000 |  |
| **Pooled** | Occurrence (ψ) | (Intercept) | -0.273 | -2.387 | 1.800 | 1.001 | **0.952** |
|  |  | **HAND Mean (1 km)** | **2.223** | **0.587** | **4.018** | **1.000** |  |
|  |  | **Edge Density Floodplain (300m)** | **-2.390** | **-4.380** | **-0.493** | **1.004** |  |
|  |  | Percent Cover Transition Forest (300m) | -0.904 | -2.677 | 0.851 | 1.002 |  |
|  |  | Edge Density Transition Forest (1 km) | 0.767 | -1.430 | 3.122 | 1.003 |  |
|  |  | HAND SD (1 km)^2 | -1.024 | -2.222 | 0.078 | 1.002 |  |
|  |  | Canoy Height SD (300m)^2 | 1.073 | -0.825 | 3.214 | 1.000 |  |
|  | Detection (p) - eBird | **(Intercept)** | **-3.512** | **-4.887** | **-2.132** | **1.001** |  |
|  | Detection (p) - Audio | (Intercept) | -1.242 | -3.147 | 0.667 | 1.000 |  |
|  |  | **Day of Year** | **-1.465** | **-2.504** | **-0.452** | **1.000** |  |
|  |  | Day of Year^2 | -0.866 | -2.295 | 0.553 | 1.001 |  |
|  |  | **Hours of Day^2** | **2.273** | **0.746** | **3.975** | **1.004** |  |

All predictor scores are logit-scaled and unitless. Bolded predictors are significant (95% BCI does not include zero). The best-performing model for this species was the pooled model, which showed that site occupancy was positively associated with HAND and negatively associated with edge density of floodplain. The audio-only model performed slightly less well, but also indicated positive associations between site occupancy and HAND. The eBird-only model performed substantially worse than either of the other models and did not include any strongly informative predictors.

**4. Occupancy model parameter estimates, *M. campanisona***

| **Model** | **Process** | **Model Covariates** | **Est.** | **Lower 95% BCI** | **Upper 95% BCI** | **Rhat** | **PR-AUC** |
| --- | --- | --- | --- | --- | --- | --- | --- |
| **Audio** | Occurrence (ψ) | (Intercept) | -0.425 | -2.088 | 1.270 | 1.000 | **0.748** |
|  |  | **HAND Mean (300m)** | **2.392** | **0.600** | **4.205** | **1.000** |  |
|  |  | Canopy Height Mean (300m) | -1.020 | -3.043 | 0.879 | 1.000 |  |
|  |  | Edge Density Open Water (300m) | -0.993 | -3.561 | 1.734 | 1.001 |  |
|  |  | Percent Cover Open Water (300m) | -0.734 | -3.551 | 1.932 | 1.001 |  |
|  |  | Canopy Height SD (1 km) | -1.193 | -3.064 | 0.586 | 1.001 |  |
|  |  | Edge Density Open Water (1 km) | 0.927 | -0.957 | 2.711 | 1.000 |  |
|  |  | HAND SD (1 km)^2 | -1.328 | -2.848 | 0.056 | 1.000 |  |
|  | Detection (p) | (Intercept) | -0.576 | -1.809 | 0.557 | 1.000 |  |
|  |  | Hours of Day | -1.233 | -3.768 | 1.622 | 1.000 |  |
|  |  | Effort Hours | 0.655 | -0.233 | 1.491 | 1.002 |  |
|  |  | Hours of Day^2 | 1.658 | -0.305 | 3.812 | 1.002 |  |
| **eBird** | Occurrence (ψ) | (Intercept) | -1.901 | -4.126 | 0.467 | 1.003 | **0.455** |
|  |  | Percent Cover Terra Firme (1 km)^2 | 1.060 | -0.109 | 2.703 | 1.002 |  |
|  | Detection (p) | (Intercept) | -1.521 | -3.137 | 0.089 | 1.001 |  |
|  |  | Day of Year^2 | -1.380 | -3.018 | 0.150 | 1.002 |  |
| **Pooled** | Occurrence (ψ) | **(Intercept)** | **-2.693** | **-4.107** | **-1.273** | **1.000** | **0.670** |
|  |  | Edge Density Open Water (300m) | -0.976 | -2.421 | 0.388 | 1.000 |  |
|  |  | Canopy Height Mean (1 km) | 0.861 | -0.880 | 2.686 | 1.000 |  |
|  |  | **Canopy SD (1 km)^2** | **1.277** | **0.223** | **2.590** | **1.000** |  |
|  | Detection (p) - eBird | (Intercept) | -1.363 | -2.777 | 0.045 | 1.001 |  |
|  |  | Effort Hours^2 | -1.076 | -3.420 | 1.082 | 1.000 |  |
|  | Detection (p) - Audio | (Intercept) | -0.292 | -1.497 | 0.864 | 1.000 |  |
|  |  | Hours of Day | -1.284 | -3.974 | 1.394 | 1.000 |  |
|  |  | **Effort Hours** | **1.257** | **0.441** | **2.052** | **1.001** |  |
|  |  | Hours of Day^2 | 1.717 | -0.323 | 3.855 | 1.002 |  |

All predictor scores are logit-scaled and unitless. Bolded predictors are significant (95% BCI does not include zero). The best model for this species was the audio-only model, which showed that site occupancy was positively associated with HAND. The pooled model performed somewhat worse, and showed that site occupancy was positively associated with extreme values of canopy height SD. The eBird-only model performed substantially worse than either of the other models and did not include any strongly informative predictors.

**5. Occupancy model parameter estimates, *O. salvini***

| **Model** | **Process** | **Model Covariates** | **Est.** | **Lower 95% BCI** | **Upper 95% BCI** | **Rhat** | **PR-AUC** |
| --- | --- | --- | --- | --- | --- | --- | --- |
| **Audio** | Occurrence (ψ) | **(Intercept)** | **3.383** | **1.694** | **5.157** | **1.001** | **0.923** |
|  |  | Canopy Height Mean (1 km)^2 | -0.709 | -2.216 | 0.882 | 1.003 |  |
|  |  | Percent Cover Transition Forest (1 km)^2 | -0.357 | -1.303 | 0.854 | 1.006 |  |
|  | Detection (p) | (Intercept) | -1.017 | -2.184 | 0.081 | 1.000 |  |
|  |  | **Hours of Day** | **-3.261** | **-5.129** | **-1.308** | **1.001** |  |
|  |  | Effort Hours | 0.365 | -0.037 | 0.765 | 1.001 |  |
|  |  | **Day of Year^2** | **0.815** | **0.460** | **1.168** | **1.001** |  |
|  |  | Hours of Day^2 | 1.507 | -0.775 | 4.032 | 1.000 |  |
| **eBird** | Occurrence (ψ) | (Intercept) | 0.105 | -1.857 | 2.293 | 1.001 | **0.844** |
|  |  | Canopy Height Mean (1 km) | 1.430 | -0.444 | 3.560 | 1.001 |  |
|  |  | Hand Mean (1 km)^2 | -1.880 | -4.392 | 0.145 | 1.003 |  |
|  | Detection (p) | **(Intercept)** | **-2.381** | **-3.428** | **-1.219** | **1.000** |  |
|  |  | Effort Hours | 0.493 | -0.073 | 1.043 | 1.000 |  |
| **Pooled** | Occurrence (ψ) | (Intercept) | -0.546 | -1.955 | 0.935 | 1.001 | **0.948** |
|  |  | Canopy Height Mean (1 km) | 2.089 | -0.025 | 4.363 | 1.001 |  |
|  |  | **Edge Density Floodplain (300m)^2** | **1.787** | **0.011** | **3.735** | **1.001** |  |
|  |  | Percent Cover Floodplain (1 km)^2 | 1.388 | -0.038 | 3.062 | 1.003 |  |
|  | Detection (p) – eBird | **(Intercept)** | **-3.262** | **-4.266** | **-2.325** | **1.000** |  |
|  |  | Hours of Day | -0.278 | -1.203 | 0.566 | 1.001 |  |
|  |  | Effort Hours | 0.249 | -0.379 | 0.850 | 1.001 |  |
|  |  | Number of Observers | 0.539 | -0.116 | 1.256 | 1.001 |  |
|  | Detection (p) – Audio | (Intercept) | 0.632 | -0.612 | 1.947 | 1.000 |  |
|  |  | Day of Year | -0.143 | -0.585 | 0.256 | 1.000 |  |
|  |  | **Hours of Day** | **-2.809** | **-4.701** | **-1.049** | **1.000** |  |
|  |  | Hours of Day^2 | 1.647 | -0.785 | 4.036 | 1.000 |  |
|  |  | Effort Hours^2 | -0.752 | -1.529 | 0.102 | 1.002 |  |

All predictor scores are logit-scaled and unitless. Bolded predictors are significant (95% BCI does not include zero). The pooled model was the best model for this species and indicated that site occupancy is positively associated with extreme values of floodplain edge density. The audio-only model showed only weak associations of occupancy probability with any predictor, despite performing fairly well. The eBird-only model had lower performance than either of the other models and did not include any strongly informative predictors.
