# Supplementary material for: Combining acoustic survey and citizen science data yields enhanced species distribution models for tropical rainforest birds
Source: PLoS One. 2025 Jul 8;20(7):e0327944. doi: 10.1371/journal.pone.0327944 (PMC12237072; doi:10.1371/journal.pone.0327944)
Supplement: Attachment — Submitted filename: PONE-D-24-56728-review_comments.pdf [file pone.0327944.s022.pdf]

In this study, the authors present a novel method for fusing citizen science eBird observations with acoustically derived bird species data to generate species distribution maps in southeastern Peru. Overall, the manuscript is very well constructed, with a thorough introduction, methods, and exploration of results. The methods section repeats itself at points, and the length of the manuscript with multiple modeling sections can be difficult to follow at times. The discussion also expands beyond the findings presented in the results section without connecting the dots of what was found here and how this applies more broadly. However, the study is intriguing and a great addition to the ecoacoustic research community.

I have organized my comments by major and minor points, which I have labeled with line numbers and figure numbers. Similar topics are grouped as well.

Large comments:

1. Occupancy modeling
    a. Results/Fig6: why is only the % difference in eval metrics presented and not the actual final model performance for each species? This makes much of the strength of models in the discussion difficult to reference.
    b. Fig7: O Salvini map is wrong relative to the supplement; is a copy of M campanisona
    c. Table S3: Metrics (WAIC?) should be defined in the table or caption. The caption is also not intuitive, specifically in saying that "For pooled models.... datasets are broken into separate rows...." which does not appear to be true.
    d. Why are standard deviation maps or other error maps not provided but they are for the encounter models? The statements throughout the discussion revolve around the ability of eBird vs audio datasets in the pooled models to fill gaps across different biomes, but without accuracy metrics or an interpretation of spatial confidence, saying that eBird has good performance in river corridors is not clearly presented. A significant amount of modeling has been done here, but the results are not linked to the discussion at a granular level before expanding to larger impacts. It is difficult to interpret whether different occupancy models are performing well or not across the landscape beyond fitting a rough a priori idea of the anticipated (binary) pattern.
    e. However, it is great to see larger-scale interpretations in the discussion!
2. 458-460: Is this for a 300m radius? That may be quite large for perfect detectability unless these species are extremely vocal and calls do not quickly attenuate in the 300m radius (especially behind a recorder). Later in the discussion, it is mentioned that tropical ARUs can be placed as close as 150-200m apart, which suggests that a 300m radius may actually not be a perfect detection radius (i.e., the ARUs may be missing detections beyond 150-200m).

Minor comments/suggestions:

1. Fig 1:
    a. 1d: Based on the LULC classification, what I expect to be sandbars are classified as built areas (e.g., at the inner elbow of river bends) in the inset. A more distinctive color ramp would aid in this interpretation, but if these are classified as built areas, that makes use of % Anthropogenic questionable, and the validity of the LULC map is less certain, especially without a statement acknowledging its limitations.
    b. The color ramp in A is different than B.

    c. It would be useful to have the equivalent regional inset mentioned in Figure 5 representing the urban gradient.

2. 34-40: long sentences, may be worth splitting this up with specific findings referenced. Also the authors propose a fused eBird/audio solution to span ecological gradients. Would an audio-only dataset that captures the "response to anthropogenic disturbance" achieve this? So, is the important finding that the eBird data enables improved generalization because of its large spatial and temporal footprint relative to audio datasets, as is presented in the introduction and not it's bias towards anthropogenic-dominated spaces?
    a. 709: an interpretation/result related to different anthropogenic pressures was not presented. For example, is there a tendency for eBird sites to span multiple LULCs within the 300m radius that include anthropogenic impact? They do span anthropogenic use spaces, but does the training dataset that results from filtering still reflect this gradient?
3. 100: typo after "data pooling…"
4. 119-120: A comparison of performance to temperate systems was not explicitly done.
5. 223: Model iii description needs to be clarified. E.g., model 3 is a model with environmental covariates... to account for X. The environmental covariates are…
6. 241: was there any field support/other work suggesting that 1 hour is considered an appropriate window to capture an individual?
7. 249-50: The joining of pres/abs data to wav files seems unintuitive as the pres/abs were determined in 1-hour intervals using the logistic GAM, but the WAVs are sometimes 24 hr continuous, intermittent continuous, or 15min. Does this result in a pres/abs associated with each observation representing 1 hour of acoustic data (e.g., WAV files are split in the table) or assigned a single observation?
8. 257-260: I have not prepared eBird observational data and only used pre-processed datasets. Are the parameters in lines 257-260 standard practice for determining sampling effort?
9. 291: The global CH map is a fusion of GEDI lidar and Sentinel 2
10. 291-292: worth mentioning the resampling technique for LULC
11. 293-304: Nice method accounting for the region of applicability!
12. 356: is there concern that calibrating the encounter model with training data may result in overfitting/overconfidence?
13. 424: specify the extent/buffer/region this % summarizes, is it still 300m radius? Also the phrasing from the prior sentence to this regarding what variables are present in each model class is confusing.
14. Fig4/526-527: not clear what this is trying to say.
15. 537-39: Very interesting result that the pooled model balances the strengths/weaknesses this well!
16. 623-624: the South Asia studies may be more densely sampled by eBird than the tropics as a whole, but does this region fall in the same category?
17. 644: typo at the beginning of the sentence.
18. S7/S9/S10 but not S8/S11: there is quite significant NorthSouth banding in the audio model and to a lesser extent in the pooled around 69.0W, and ~69.9W. The eBird model shows this in S10. Is there a reason why this is showing (e.g., variable processing)?
19. S13: add x-axis unit label
20. S12/13: descriptions are not included on page 52.
