# Supplementary material for: Combining acoustic survey and citizen science data yields enhanced species distribution models for tropical rainforest birds
Source: PLoS One. 2025 Jul 8;20(7):e0327944. doi: 10.1371/journal.pone.0327944 (PMC12237072; doi:10.1371/journal.pone.0327944)
Supplement: Attachment — Submitted filename: Response to Reviewers.docx [file pone.0327944.s024.docx]

**Manuscript PONE-D-24-56728
Response to Reviewers**

Dear Dr. Jones,

Thank you for allowing us to submit a revised draft of the manuscript titled “Combining acoustic survey and citizen science data yields enhanced species distribution models for tropical rainforest birds” for consideration in *PLOS One*. We appreciate the time you and the two academic reviewers have dedicated to reading our manuscript draft and are grateful for the feedback we have received, which we have incorporated into our revised manuscript. In addition to improving the overall quality of writing, we have made substantial alterations to our modeling framework to account for differences in spatial specificity between our datasets, and have replaced qualitative statements about model performance with more quantitative statistical tests wherever possible. We believe both of these changes have improved the scientific rigor and generalizability of our analysis.

Please see below for an itemized response to each reviewer’s comments. Line numbers referred to in these comments apply to the original version of the manuscript. In addition, I have attempted to reorder the comments from reviewer 1 so that they are in the same order as the referenced lines / figures / tables occur in the manuscript.

Many thanks,

Reid Rumelt

Ph.D. Candidate

Conservation Ecology Lab | Dr. Chris Searcy

Department of Biology | University of Miami

rumeltr@miami.edu

**Responses to Reviewer 1:**

Authors state data will be made available after review otherwise my comments are included in the attached pdf (copied below). This is a very interesting study that I feel will be relevant for the ecoacoustic community.

34-40: long sentences, may be worth splitting this up with specific findings referenced.

*Agreed, I have broken up the sentences at the end of the abstract and have tried to* *integrate more specific information about the analysis’s main findings.*

34-40: Also the authors propose a fused eBird/audio solution to span ecological gradients. Would an audio-only dataset that captures the "response to anthropogenic disturbance" achieve this? So, is the important finding that the eBird data enables improved generalization because of its large spatial and temporal footprint relative to audio datasets, as is presented in the introduction and not it’s bias towards anthropogenic-dominated spaces?

*The primary way in which eBird data are biased towards anthropogenic areas is its broader spatial coverage as, in the current study, there is zero audio data in those* *areas. An audio dataset that captured “response to anthropogenic disturbance”* *would absolutely help achieve a similar result… fact, I am currently in the process of* *collecting similar acoustic data along the Manu road and would conceivably be able* *to try this out in that system! The hard part is collecting that data due to permitting, the need to adequately cover the different types of disturbed areas, and the person hours needed for recorder maintenance. eBird data provides this information, perhaps slightly less well and at a lower resolution than acoustic data, but in a form that is far more accessible to researchers. I have edited my statement in the abstract to account for this as well as the previous comment:

“Our approach combined automated acoustic monitoring detections and eBird citizen science data available for the region as well as a high resolution land cover dataset of the region’s key ecological gradients. The pooled models outperformed models constructed solely with eBird data for predicting fine grain species responses to habitat gradients in intact forest, but also retained information from the citizen science dataset about species occurrence patterns in non-vegetated areas away from intact forest, including those subject to human disturbance. We present this hybrid approach as a flexible and repeatable means to produce inferences that would not easily be achievable using a single data source, and provide recommendations for other researchers seeking to replicate these methods in Amazonia as well as in other tropical regions.”*

Fig 1: Based on the LULC classification, what I expect to be sandbars are classified as

built areas (e.g., at the inner elbow of river bends) in the inset. A more distinctive

color ramp would aid in this interpretation, but if these are classified as built areas,

that makes use of % Anthropogenic questionable, and the validity of the LULC map

is less certain, especially without a statement acknowledging its limitations.

*This is why being able to ground-truth remote sensed imagery is so important. Your reading of the LULC color ramp is accurate, those areas are classified as built… and concerningly this is because remote sensing does a surprisingly good job of highlighting human disturbance in areas where you might not expect there to be, at least if you’ve never traveled to this part of Peru. As of the time the satellite data were captured, most of the things that look like sandbars near Los Amigos are actually dredge tills from alluvial gold mining operations. Many of these also have on them machinery as well as small structures where the miners live, and one of these two things is causing the LULC to classify these areas as “built.” I don’t think this distinction changes the results of the analysis much, as none of these interior forest birds should occur with any frequency in the non-forest habitat types and I have confirmed that all of these tills are > 1 km from the acoustic points so they never have non-zero values for that dataset. However, I am going to add some text after line 287 to this effect:*

*“This land cover layer includes several non-forest land categories (such as bare ground, crops, and built areas); while none have non-forest values at the acoustic points (preliminary data analysis indicates that all of the acoustic points are ≥ 1 km from any non-forest land cover classes), we believed they would be crucial for measuring absences for eBird sites in non-forest areas. Ground-truthing of the Madre de Dios corridor also indicates that dredge tills and temporary habitations on river beaches subject to alluvial gold mining [15] are typically (correctly) classified as “built,” and we felt including these categories might also help our models capture the effects of this form of disturbance on species presence at eBird sites along rivers.”*

Fig 1: The color ramp in A is different than B.

*Good catch… this is fixed in the resubmission.*

Fig 1: It would be useful to have the equivalent regional inset mentioned in Figure 5

representing the urban gradient.

*Added in the resubmission. That being said, I want to make clear that this analysis considers forest and anthropogenically modified habitats as a binary rather than a gradient.*

Line 100: typo after “data pooling…”

*Fixed.*

Lines 119-120: A comparison of performance to temperate systems was not explicitly done.

*We never intended to do this, and instead opted to compare our results in a qualitative sense to work done in the temperate zone by other researchers. I have altered this sentence to avoid suggesting that we intend to do an explicit comparison in this study:

“We aim to evaluate whether this data pooling approach, combined with a higher resolution land cover dataset of the key ecological gradients in this region, is capable of producing inferences that are not possible with models using solely acoustic or solely eBird data. In addition, we test whether data pooling improves the ability of models to capture local-scale habitat preferences in tropical forest, as has previously been shown to be true for temperate systems [42].”

I am also adding a reference to the Robinson et al. paper as an example of a temperate zone study where a similar approach was used.*

Line 223: Model iii description needs to be clarified. E.g., model 3 is a model with environmental covariates... to account for X. The environmental covariates are…

*I have edited the text of this description to make the model structure more clear:

“iii) a logistic generalized additive model (family “binomial” with REML optimization) with logit score (linear term) as a predictor and label as the response, but with the addition of site elevation (smooth term, cubic regression spline) as an environmental covariate to account for the possibility that classifier performance might vary across the habitat gradient due to the presence of similar-sounding organisms across certain sections of the gradient”*

Line 241: was there any field support/other work suggesting that 1 hour is considered an

appropriate window to capture an individual?

*For some reason a reference to research conducted by coauthor Carla Mere in which she used 1 hour windows in her work on tinamou vocalizations at Los Amigos didn’t make it into the original submission, so I am including those now.*

Lines 249-50: The joining of pres/abs data to wav files seems unintuitive as the pres/abs were determined in 1-hour intervals using the logistic GAM, but the WAVs are sometimes 24 hr continuous, intermittent continuous, or 15min. Does this result in a pres/abs associated with each observation representing 1 hour of acoustic data (e.g., WAV files are split in the table) or assigned a single observation?

*I agree that this section could have been clearer. The way the pseudoreplication filtering worked in practice was that vocal events were assigned to the specific hour they were recorded in. Every detection of the same species / recorder / day received an integer corresponding to the hour when the detection occurred and we saved a single detection per species per hour per recorder. I have edited this section to clarify that this is actually what’s happening:

“To facilitate joining acoustic and eBird data for use in model creation, we formatted the acoustic detection CSV file so that it contained the same columns as the eBird dataset download. Using the ‘auk’ package in R [81], we compared the detection set to a database that contained a list of all one-hour recording periods across the field seasons with associated site-date information. Each hour-long recording period received a 1 for each of the target species that was present and 0 for each of the target species that was absent. We hereafter refer to this type of presence-absence dataset taking into account all sampling events as “zero-filled,” as this is the term used in the literature on eBird SDM [82].”*

Lines 257-260: I have not prepared eBird observational data and only used pre-processed

datasets. Are the parameters in lines 257-260 standard practice for determining sampling

effort?

*Most of these values are consistent with what is typically used in the literature on*

*eBird SDM, though we reduced the checklist length cutoff to 1 km (from 5 km) to*

*account for the fact that habitat gradients in the tropics tend to be shorter than in*

*the temperate zone. I have added some text explaining that this is the case:

“To further increase sampling effort evenness within the dataset, we filtered this dataset to contain only complete checklists (those where the observer specifies that they reported all species they were able to identify) with survey duration less than five hours, effort distance less than 1 km (for traveling checklists), and number of observers less than 5. While these filtering constraints are broadly consistent with what is typically used in the literature on eBird SDM [82], our shorter distance cutoff (1 km as opposed to 5 km) accounts for the fact that ecological gradients in the Tropics tend to be shorter than in the temperate zone [83].”*

Line 291: The global CH map is a fusion of GEDI lidar and Sentinel 2

*Thanks for the clarification, I have added this information to the text.*

Line 291-292: worth mentioning the resampling technique for LULC

*Added. We used nearest neighbor for categorical data, bicubic for continuous data.*

Lines 293-304: Nice method accounting for the region of applicability!

*Thanks!*

Line 356: is there concern that calibrating the encounter model with training data may result in overfitting/overconfidence?

*Using a constrained GAM is recommended as a calibration method in the literature on eBird SDM as it reduces the chance of overfitting the model given that training data is being used (see Strimas-Mackey et al.’s “eBird Best Practices” guide). I have added text to this effect in the section you highlight here:

“Using a constrained GAM is recommended as a calibration method in the literature on eBird SDM as it reduces the chance of overfitting the model given that the calibration is being performed using the training data [18,100,101].”*

Line 424: specify the extent/buffer/region this % summarizes, is it still 300m radius? Also the phrasing from the prior sentence to this regarding what variables are present in each model class is confusing.

*Yes, this was calculated within the same 300m radius buffers that we used for all data points throughout the submission you reviewed. Although much of this passage has been rewritten in the resubmission as a result of comments from reviewer 2, I have nonetheless tried to reword this sentence to make it more clear that we are doing this for the same buffer size(s) used earlier in the analysis:*

*“We calculated the amount of anthropogenic habitat within each buffer size class as the sum of “crop” and “built area” land cover types as these are the two classes that are unambiguously the result of human influence. This accounts for residual differences in occurrence due to human land use that are not explained by canopy height alone.”

In the resubmission, the prior sentence about which variables served what roles in the model has been replaced with the following sentence in the second paragraph of the occupancy modeling section:

“Based on our exploratory data analysis and an examination of preliminary results from the encounter rate modeling component, we allowed audio-only and pooled models to contain habitat predictors from both 300 m and 1 km spatial scales, while only including 1 km predictors in the eBird-only models. For detection covariates, we allowed the eBird-only models to contain a complement of typical eBird checklist effort covariates (day of year, hours of day, effort hours, protocol type (stationary or traveling), effort distance (km), and number of observers). For the audio-only models, we included the subset of these covariates that are relevant to ARU data (day of year, hours of day, and effort hours). To account for the fact that site-surveys represent multiple days in the ARU data, we defined*

*hours of day as representing the hour in which the first detection of the given species occurred during a sampling period, and effort hours as the total number of hours during each three day survey period that the recorder was turned on.”*

Lines 458-460: Is this for a 300m radius? That may be quite large for perfect detectability unless these species are extremely vocal and calls do not quickly attenuate in the 300m radius (especially behind a recorder). Later in the discussion, it is mentioned that tropical ARUs can be placed as close as 150-200m apart, which suggests that a 300m radius may actually not be a perfect detection radius (i.e., the ARUs may be missing detections beyond 150-200m).

*The statement about perfect detectability applies within the buffer representing the zone of acoustic detectability (the 150m cone around each recorder in which birds are able to be recorded) rather than the 300m buffer within which the habitat variables are summarized. These are not meant to be the same in this analysis, and we feel there is merit to having habitat summaries extend beyond the audible cone of detection. Birds, even those that occur in rainforest understory, do move around the landscape during the course of their lives, and it is also likely that birds on the edge of the cone of detectably would still respond to edge effects or ecotones even if they fall somewhat outside of this cone. Having a wider cone better describes the habitat that the birds being recorded can access. In fact, based on feedback from reviewer 2, I am now adding an additional set of summary variables within 1 km buffers in addition to the 300 m buffers I was using previously. However, I did some exploratory data analysis with the acoustic sites and it turns out that when you calculate the cumulative detection probability directly using the audio-only occupancy model, some sites where less recording was done do indeed have p<0.9. As a result, I am now filtering these sites similarly to the eBird sites instead of assuming p~1. This sentence now reads:

“Although acoustic sites had substantially higher sampling intensity on average than the eBird sites (mean total effort-hours of 176 per site versus 3.6 for the eBird sites), exploratory data analysis nonetheless showed that cumulative detectability at a small number of acoustic monitoring sites with the shortest total recording durations was ≤ 0.9.”*

Lines 526-527 / Fig 4: not clear what this is trying to say.

*I agree with you that this figure (and likely the general approach of describing these changes qualitatively) is not particularly easy to follow. For reference, the context of the encounter rate analysis, habitat predictors (in blue in these and other figures) are those that speak to species habitat affinities, whereas those in gray describe how differences in survey effort influence detection. Rather than calling certain predictors “salient” and describing them qualitatively, in the revised manuscript I am now designating habitat predictors that are relevant to the short spatial scale habitat gradients present within intact forest (percent land cover types, edge density, and HAND) and test quantitatively / statistically whether they are given a higher rank by one or more model classes than canopy height (and of course also land cover types in non-forest areas), which better distinguishes forested from non-forested sites than it does the various forested sites. For the resubmission, (lines 370-387), this section has been changed to the following (apologies for the length… important to explain all changes for context):*

*“Finally, we used ‘ranger’ to calculate relative predictor importance for each model using the Gini Score [104], where relative importance value is calculated based on the mean decrease in model accuracy when the chosen predictor is excluded during the fitting process [82]. Our inclusion of predictors from both 300 m and 1 km spatial scales allowed us to directly test whether one spatial scale was preferred over another in each of the model classes. This was done by fitting a linear mixed-effects model [105] with importance score as response, predictor and the full interaction between distance and dataset as fixed effects, and species as a random effect. We also used these scores to test two predictions about relative importance shifts of habitat predictors between model classes. First, as we view the encounter rate metric to be the product of a “signal” (the occurrence process) which we care about and “noise” (the detection component) that we wish to ignore, we value models in which habitat predictors are assigned high ranks relative to detection covariates. As the acoustic dataset has more consistent levels of survey effort than the eBird dataset, we predicted that habitat predictors would be ranked more highly relative to detection covariates in the pooled models than in the eBird-only models. We tested this prediction using a linear mixed-effects model with importance as response, predictor as a fixed effect, the full interaction of “is habitat predictor” (categorical, yes or no) with dataset as a second fixed effect, and species as a random effect. Second, as we felt acoustic data provide more specific information than eBird data about where species occur within intact forest, we predicted that certain forest characteristic indicators (FCIs) should be ranked higher in importance relative to predictors measuring occurrence differences between forest and non-forest areas in the pooled model class compared to the eBird-only model class. We designated HAND and percent cover of floodplain, transition forest, and terra firme as our FCIs: HAND is the single strongest proxy of forest character in this region as flooding disturbance frequency follows an elevation gradient away from major rivers [58], and the two land cover variables are both very strongly correlated with HAND (r ≥ 0.8). As such, we feel species responses to these predictors should strongly indicate in which part of the intact forest landscape they occur. By contrast, we considered canopy height (not strongly correlated with HAND, r ~ 0.4), percent cover crops, percent cover built area, and percent cover bare ground to be more relevant to differences in species occurrence between forest and non-forest areas. We assessed this relationship using a linear mixed-effects model with importance as response, predictor as a fixed effect, the full interaction of “is FCI” (categorical, yes or no) with dataset as a second fixed effect, and species as a random effect.”

I also now describe the results of these tests in the Discussion section. The summarized version is that the key interior forest predictors are *not* ranked higher in any of the model classes relative to any of the others, which surprised me, though the eBird-only models rank that second set of predictors higher relative to the audio-only models, but the audio-only and pooled models ranked them about the same.*

537-39: Very interesting result that the pooled model balances the strengths/weaknesses this well!

*Thanks!*

Results/Fig 6: why is only the % difference in eval metrics presented and not the actual final model performance for each species? This makes much of the strength of models in the discussion difficult to reference.

*The primary reason I chose to represent this as a set of percentages was to make it easier to compare results between this figure and figure 3. However, I see your point, and seeing as I already do this for the encounter rate analyses, I have added a figure (new Fig 7, bumping the occupancy maps to Fig 8) to show the raw occupancy performance metrics as well.*

Fig 7: *O Salvini* map is wrong relative to the supplement; is a copy of *M. campanisona.*

*Yup, thanks for pointing that out… it was a typo in the code, and it’s fixed in the resubmission.*

Fig 7: Why are standard deviation maps or other error maps not provided but they are for the encounter models? The statements throughout the discussion revolve around the

ability of eBird vs audio datasets in the pooled models to fill gaps across different

biomes, but without accuracy metrics or an interpretation of spatial confidence,

saying that eBird has good performance in river corridors is not clearly presented. A

significant amount of modeling has been done here, but the results are not linked to

the discussion at a granular level before expanding to larger impacts. It is difficult to

interpret whether different occupancy models are performing well or not across the

landscape beyond fitting a rough a priori idea of the anticipated (binary) pattern. However, it is great to see larger-scale interpretations in the discussion!

*I have updated the figures in the supplement to include a panel for each model with the occupancy model SE.*

Lines 623-624: the South Asia studies may be more densely sampled by eBird than the tropics as a whole, but does this region fall in the same category?

*The two regions are less comparable than it might seem. To illustrate: the most heavily eBirded region of Peru is Cuzco Department, which is 71,986 km^2^ and has ~89,900 eBird checklists (~1.25 checklists/km^2^) as of March 2025. Madre de Dios, the region where this study was conducted, is just barely smaller at 85,300.54 km^2^ but has almost half as many checklists (48,000, or ~0.56 checklists/km^2^). Meanwhile, Kerala, the most eBirded state in India, is smaller than both of them (38,863 km^2^) but has 664,800 checklists (17.1 checklists/km^2^). The second study took place in Tamil Nadu, which is 130,058 km^2^ and has ~498,400 checklists (3.83 checklists / km^2^) Arunachal Pradesh, where the first study was conducted, is a bit more comparable at 83,743 km^2^ and 60,671 checklists, but it still has a higher level of sampling intensity than Madre de Dios. I have added a more succinct version of these stats to the discussion section to back up the statements I’m making.*

Line 644: typo at the beginning of the sentence.

*Fixed.*

Line 709: an interpretation/result related to different anthropogenic pressures was not

presented. For example, is there a tendency for eBird sites to span multiple LULCs

within the 300m radius that include anthropogenic impact? They do span

anthropogenic use spaces, but does the training dataset that results from filtering still

reflect this gradient?

*I think this statement was left over from before I decided to lump the different anthropogenic categories together for the occupancy models, which is why it was never treated in the text. However, it occurs to me that this would be very easy to accomplish by looking at the full list of predictor importance scores in the encounter rate models (e.g. not just the top 10 that are in the figures). I have added a table summarizing the importance scores of the anthropogenic land cover classes and the following text after line 520:

“Having access to the predictor importance scores also allowed us to assess the negative contributions of different anthropogenic habitats to predicted occurrence of our target species. Among the various land cover types we use as predictors in the encounter rate analysis, we consider two of these, percent cover built area and percent cover crops, to be unique signals of anthropogenic disturbance. Although neither were ranked high enough to be included in the top 10 most important predictor variables for any species (S6 Fig), the former was the highest ranked “anthropogenic” land cover class in all species in both eBird and pooled model types (Table 2).”*

Table S3: Metrics (WAIC?) should be defined in the table or caption. The caption is

also not intuitive, specifically in saying that "For pooled models.... datasets are

broken into separate rows...." which does not appear to be true.

*I have edited the caption to include the definition for WAIC. The sentence in the caption that was confusing you pertained to how an older version of this table was formatted, but when the table was changed to the current version it seems that text was never removed, so I have done so now.*

Fig S7/S9/S10 (but not Fig S8/S11): there is quite significant North/South banding in the audio model and to a lesser extent in the pooled around 69.0W, and ~69.9W. The eBird model shows this in S10. Is there a reason why this is showing (e.g., variable processing)?

*The banding you see is an artifact in the canopy cover layer, and appears more strongly in species where that was an important predictor variable in the model. I’m not sure whether this is something in the GEDI data or is left over from when they stitched together the different Sentinel-2 images, as the canopy cover raster was a data product that I downloaded from the cited source (rather than building myself) and this artifact was present in the download.*

Fig S13: add x-axis unit label

*Fixed.*

Fig S12/13: descriptions are not included on page 52.

*Added.*

**Responses to Reviewer 2:**

Introduction: This section presents clear background information on the methodologies, identifies a deficiency of this type of work in the neotropics, and states the intentions of the study. The narrative flow is generally good and easy to follow, but I suggest several considerations below.

Lines 59-64: This paragraph seems out of place to me. It interrupts the flow from the identification of increasing data availability and innovation of new tools in the first paragraph to the introduction of ARUs as tool to be used in the tropics. It would make sense to include this type of study region information at the beginning of the last paragraph in the Introduction as a way to further justify your efforts.

*I have moved this paragraph so that it is the second to last paragraph in the introduction section and have modified it so that it does a better job of setting up the description of what we did in the paper:

“The Amazon rainforest is one of the most speciose regions for birds globally [45,46]. Major drivers of diversity in this region include rivers, other habitat discontinuities [47–49], and interspecific competitive interactions [50,51], but also habitat heterogeneity and long-term ecological stability [52–55]. Using novel modeling frameworks to assess how habitat gradients mediate bird occurrence patterns could help answer important questions about community assembly processes and habitat partitioning within clades of Amazonian birds that up to this point have been difficult to answer due to data and survey effort limitations.”*

Line 67: You say that ARUs are more effective than humans at detecting birds in dense understory conditions but later contradict this idea in the Discussion by acknowledging the benefits of human observers.

*You’re right, I do contradict this statement in the Discussion. I am removing the “or human observers” from this sentence so as not to detract from the statements I make in the Discussion about the benefits / drawbacks of acoustic monitoring relative to visual / aural encounter surveys.*

Lines 68-71: This is fine information to include (e.g. justification of ARU usage in the tropics), but throughout the remainder of the paper conservation/survey urgency in relation to this work is not mentioned again so I wonder how beneficial it is here.

*In the last paragraph I circle back around to the topic of conservation urgency by describing specific anthropogenic disturbance forces affecting MDD. However, if you weren’t able to draw links between the two passages then it is my responsibility to make it more obvious that this is my goal. I have altered the referenced lines to read:

“The ability of automated recording devices to collect large volumes of data at specific sites also makes this survey technique a critical tool in areas that are likely to experience significant anthropogenic disturbance in the near future and for which limited time exists to gather data [14–16].”

I have also edited the last paragraph to include information about why specific aspects of ARU recording are beneficial in the context of anthropogenic change:

“We have demonstrated here that ARU surveys combined with machine learning acoustic classifiers can be used to rapidly collect occurrence datasets for interior forest birds; when used as part of an integrated modeling framework, this data can be used to model species habitat associations across habitats. In addition to helping researchers build a more complete picture of Amazonian diversity, such inferences will also be critical for predicting responses to anthropogenic land use change, which is a key research priority given rapid regional increases in deforestation, fire, road-building [63,128,129] and illegal artisanal gold mining along riverine corridors [64,65]. Understanding species’ current patterns of habitat utilization will be crucial for understanding potential responses to various anthropogenic pressures as well as helping to inform conservation efforts and acquisition of land for the establishment of protected areas.”*

Lines 76-81: These two sentences duplicate ideas. I’d suggest condensing them into one consistent idea.

*I have condensed these lines into the following sentence:

“However, while automated recording units (ARUs) are capable of recording continuously for many days or weeks given sufficient battery and storage capacity, the need to purchase several units given the small area of coverage of each (~150 m radius around the recorder [21]), and the field labor required for servicing means that monitoring projects using ARUs are typically focused on high density of sampling sites rather than wide spatial coverage [22].*

Line 92: spell out MODIS

*Done.*

Line 100: “data pooling has been increased the…” change to “data pooling has increased…”

*Done.*

Lines 119-120: While curiosity is an important component of this research, I think rephrasing this thought to something more direct and objective-like would be better for this section.

*I have revised these lines to the following:

“We aim to evaluate whether this data pooling approach, combined with a higher resolution land cover dataset of the key ecological gradients in this region, is capable of producing inferences that are not possible with models using solely acoustic or solely eBird data. In addition, we test whether data pooling improves the ability of models to capture local-scale habitat preferences in tropical forest, as has previously been shown to be true for temperate systems [42].”*

Lines 123-125: To what extent and where is this done in the manuscript? Maybe I am missing it in the discussion.

*I have added a number of points in the Discussion describing potential pitfalls of this approach that we encountered during this analysis and giving suggestions for how this methodology could be employed by others in the future. For brevity I’m not going to include them all here, but you will see them in the revised manuscript.*

Methods: Very detailed and highly repeatable although I feel that some structure is needed for readers to understand the justification and objectives of each successive step in your modeling process. At times, it is overly dense, and the overarching methodology is sometimes muddied with small side details. Some of the finer details may be best suited for the Supplemental Material section. It is also overdeveloped in comparison to other sections, especially the results and discussion. See line-by-line comments below:

Lines 184-186: I suggest the latter half of this sentence to be changed to “… for the three seasons are available in *citation 14* and in S1 File”

*I have edited the lines accordingly.*

Lines 188-196: This paragraph might fit better at the end of the methods section.

*I have moved the paragraph to the end of the Methods section, but as it was added at the request of the content editor I defer to them in the event that it needs to be moved again.*

Lines 200-204: Add brief description of study area in relation to a larger geographic area as referenced in the figure.

*I have added the following text to this figure caption: “Study region is outlined in inset map of Peru in (A).”*

Lines 216-219: I feel this and the following methodological explanation could be more clearly justified in at least one preceding sentences; some readers may be confused by the need for modeling and model selection here.

*I have edited these lines to read the following:*

*“We took a sample of the positive detections for each species spanning the full confidence score range of the classifier output (0.3-1); in order to more evenly cover the spatial gradient at Los Amigos, we stratified this sample such that up to 30 detections were chosen from each 10 m interval of elevation across acoustic sampling sites (200-293 m). Species with low average BirdNET classifier performance may have many more low confidence score detections than high confidence score detections, thus reducing sample size in the range of scores where false positives transition to true positives [78]. To account for this, we added a second independent sample of up to 30 detections at a range of higher scores (0.8-1) from each 10 m elevational band.*

Lines 239-242: This information should be stated earlier so readers are aware of this sooner.

*This step is presented in the order in which it was performed in the analysis (e.g.*

*after all other sampling effort quality filtering was performed). I feel the order in which these steps were performed is sufficiently important to a general understanding of the methods that I think it best to not move this sentence elsewhere, but I can edit it so that it makes the order of operations clearer:

“After all other sampling evenness filtering was performed, we collapsed sequences of vocal events into at most one detection per species per hour of recording time [62,80]; we refer to these as “independent detections.”*

Lines 243-250: Not major, but this entire paragraph seems like something that could easily be reduced to one sentence. Some information here does not seem critical.

*Reviewer 1 asked me to expand this section so that I made it more clear how I was treating cases where files were < 1 hr long, so I don’t think it would be a good idea for me to reduce the text down to a single sentence. These lines now read:

“To facilitate joining acoustic and eBird data for use in model creation, we formatted the acoustic detection CSV file so that it contained the same columns as the eBird dataset download. Using the ‘auk’ package in R [81], we compared the detection set to a database that contained a list of all one-hour recording periods across the field seasons with associated site-date information. Each hour-long recording period received a 1 for each of the target species that was present and 0 for each of the target species that was absent. We hereafter refer to this type of presence-absence dataset taking into account all sampling events as “zero-filled,” as this is the term used in the literature on eBird SDM [82].”*

Lines 249-250: “zero-filled” is not referenced again in the manuscript.

*I edited the text beginning on line 260 as part of my response to a comment from*

*Reviewer 1, so that the term “zero-filled” is now referenced a second time in the manuscript:

“We repeated the previously-described zero-filling process by joining the eBird checklists to the complete database of eBird checklists…”*

Lines 253-260: Why did you filter eBird data using these criteria? Are you basing this off previous work? You discuss this briefly in the Discussion, but more justification here is desired. eBird data from the tropics is notoriously biased by checklists submitted by birders from other regions who are less experienced with the local avifauna, or guides who inflate their lists in an effort to make their services more appealing. How much quality control was done on the eBird data? I think these ideas are worth addressing, if not in the Methods, then with more detail in the Discussion than is currently present. The following paper provides some structure on this process in a north temperate study: Hallman, T.A., Robinson, W.D. Supplemental structured surveys and pre-existing detection models improve fine-scale density and population estimation with opportunistic community science data. Sci Rep 14, 11070 (2024). <https://doi.org/10.1038/s41598-024-61582-6>

*There is no established set of criteria for eBird sampling effort filtering that is specific to the Neotropics (or for that matter anywhere in the Tropics), so in general I defer to those listed in Strimas-Mackey et al.’s eBird data best practices document, with the exception that I view their 5 km distance cutoff as too permissive given the shortness of ecological gradients in the Tropics. As Reviewer 1 also asked for clarity on the rationale used for choosing these filter criteria, I have edited lines 257-260 as follows:

“To further increase sampling effort evenness within the dataset, we filtered this dataset to contain only complete checklists (those where the observer specifies that they reported all species they were able to identify) with survey duration less than five hours, effort distance less than 1 km (for traveling checklists), and number of observers less than 5. While these filtering constraints are broadly consistent with what is typically used in the literature on eBird SDM [82], our shorter distance cutoff (1 km as opposed to 5 km) accounts for the fact that ecological gradients in the Tropics tend to be shorter than in the temperate zone [83].”

We did not perform any a priori eBird data quality control for this analysis beyond filtering based on the listed criteria. Our primary conclusion about the eBird data in this region is that they help establish absence patterns in places away from interior forest areas (these interior forest areas are, after all, where species are hardest to identify and where an inexperienced observer would most need the services of a guide). However, while I feel it is outside the context of this analysis to add this to the Methods at this stage, I do think the methodology in the linked paper is a neat way to approach these data quality issues and have added some text at the end of the fifth paragraph in the Discussion section suggesting that other researchers applying these methods to their systems consider using a process similar to the one described:

“Finally, it is worth keeping in mind that eBird data are collected by a variety of different observers that have varying degrees of experience with the local avifauna and follow different patterns of sampling effort allocation. One specific instance where this might have impacted our results was the case of the best-fit pooled occupancy model for O. salvini, which indicated a positive association with extreme values of floodplain edge density (S3 File) that is biologically implausible given what is known about this species’ ecological preferences [75]. It appears that a few outlier site-visits from the eBird dataset are primarily responsible for driving the shape of this relationship, all of which are checklists from popular birding guides at large, popular ecolodges (S13 Fig). Calibration indices that evaluate eBird observers using species accumulation curves are a promising strategy to account for some of this variability in birding skill level and have been employed in other similar eBird SDM analyses [39,112]. Other researchers have also found success in using statistical models to compare eBird checklist observations with point counts from experienced observers to select a subset of high quality checklists for analysis [113].”*

Lines 260-263: What are you defining as present on a checklist? What about checklists with “Xs”?

*The eBird project team do not consider “X” observations have a well-defined*

*meaning; as such, I followed the eBird best practices document’s recommendation*

*that X’s be recoded as NAs, which removes them from consideration. I have added*

*text on line 260 stating this explicitly.*

Lines 264-267: True, this is the case in north temperate eBirding communities where the majority of checklists are submitted by those very familiar with the local species, but can the same assumption be made for the neotropics?

*I chose to focus on these five species for this analysis because, among the species of interior forest birds that occur in Madre de Dios, these species are some of the easiest to make this assumption about. As far as Amazonian species are concerned, they are about as easy as it gets in terms of heard-only IDs, even for those with limited experience here. Though it is impossible to convey this verbally in my response, I invite you to listen to vocalizations for these species on the Macaulay Library or Xeno-Canto if you wish to judge this for yourself. They are also common enough that it is very difficult to go anywhere in this region during peak birding hours (in the right habitat of course) and not hear at least one or two of these species. I have added the following sentence after line 267 giving readers the ability to assess the prevalence levels of each species themselves:

“In addition, we consider our target species to be very easy to detect by eBirders: as well as having loud, simple vocalizations, they are also among the most common bird species in Madre de Dios (reported more often than 98.0% of all other species in Madre de Dios for F. analis, 87.5% for A. goeldii, 75.4% for O. salvini, 74.2% for F. colma, and 64.0% for M. campanisona).”*

Lines 273-277: This sentence could be condensed.

*Sentence is now broken into these two sentences:*

*“The MODIS land cover dataset lumps most Amazonian forest types into an “evergreen broadleaf forest” class, and has a resolution (500 m) that is too coarse to resolve the short ecological gradients present in the region [56,86,87]. As a result, we felt it necessary to develop a custom predictor dataset that is resolved to a finer spatial scale and contains more nuanced land cover classes that are relevant for analyses in the Amazon.”*

Line 275: Is “short” the right word choice here?

*The concept of “short (ecological / environmental / habitat) gradients” is a fundamental aspect of community ecology when speaking about gradients over which some aspect (or aspects) of the environment pertinent to the study species changes dramatically relative to the distance from one end of the gradient to the other. The term is used both to describe the gradient itself and to describe techniques used to summarize community composition along these gradients (such as ordinations; see the Gauch 1982 paper cited in the manuscript). I have replaced the word “habitat” with “ecological” on this line in addition to the other places where this explanation occurs in the manuscript because it seems like the latter is the more common term in the literature, but I am keeping the word “short” because this is a relevant domain concept. I am also adding references to some foundational work done on spatial scales in ecological gradients that use this term.*

Line 278: terra firme is italicized here but not consistently elsewhere is the manuscript.

*Fixed.*

Lines 316-317: Provide a citation for this sentence.

*I added two, one to the Robinson et al. 2020 analysis on shorebirds in California and the other to the Johnston et al. 2021 methods paper on eBird data.*

Line 317: “proportional” seems like not the correct word here, maybe “synonymous”?

*“Synonymous” would suggest that they are the same (or almost the same) thing, when they are in fact demonstrably different quantities. I think the disconnect here is that I need to state explicitly that I don’t mean “proportional” in a figurative sense: encounter rate is mathematically proportional to occupancy via its definition as the product of the occurrence and detectability processes (Johnston et al. 2021). However, I admit that the original sentence might not have made this fact immediately obvious to readers whose main focus isn’t mathematical modeling, so I have updated the text of this line as follows:*

*“Although it [encounter rate] is not capable of isolating the factors that determine site occupancy (z), it is nonetheless a useful approximation of this quantity as it is approximately equal to the product of the site occurrence probability (ψ) and detectability (p) processes from occupancy modeling and uses observation date, time, duration, distance covered, and number of observers as covariates in order to account for between-site variability in detection [82,96].”*

Lines 345-346: If you are including traveling checklists with < 1km distance, this buffer needs to be expanded to 1 km. When using data from eBird hotspots you should also consider the implications of extracting habitat predictor data at the coordinates of the checklist. This should be reflected here or in more detail in the discussion.

*Although the easiest way to approach this comment would probably have been to change the buffers to be 1 km across the board, this would’ve thrown away a lot of local scale inferences from the acoustic data, and I was concerned that doing so would also have led to substantial levels of spatial autocorrelation in the acoustic dataset as the 1 km buffers around recorders would have contained many other recorders. As such, I decided to instead make significant modifications to the modeling methodology, which I summarize below:*

- *I calculated 300 m and 1 km buffer summary versions of each predictor*
- *Encounter rate models were built using both versions of each predictor (I go into detail in the resubmission as to why including such a large number of predictors isn’t a big deal for randomForest models)*
- *I assess the encounter rate model results as before, but I test differences in mean Gini importance score between the two spatial scale versions of each predictor within each model class using a linear mixed-effects model*
- *Your intuition was correct in the sense that the 1 km summaries were significantly more “important” than the 300 m buffers for the eBird-only and pooled models, while the two buffer sizes were equally as important in the audio-only models. This new finding is now included in the Results section*
- *In the occupancy modeling component, I expanded the number of predictors being included in the global formula for each model (I go into greater detail on this point in my response to one of your later comments), and I only allow the global formula for the eBird-only models to include the 1 km versions of the predictors. However, I include both buffer size summaries in the pooled models since these have both ARU and eBird data in them, and in the audio models I also included both buffer size summaries as there is a (perhaps small) possibility that occupancy at the audio sites could be affected by conditions further afield than 300 m*
- *I implemented a comprehensive model selection approach (described in more detail in the resubmission) that is based on a backwards stepwise elimination process*
- *The best models after this step were used to make conclusions for the occupancy modeling component*

*These changes did not alter the overall findings of the analysis that much. However, besides satisfying your concern, I feel these modifications have also made the entire work more generalizable. I say this because this process feels more similar to what I would expect other research teams to do if their ultimate goal was to find the absolute best models for their subject species, rather than just testing a methodology, which was my goal with this manuscript. I also hope it allays your feeling that the Results and Discussion sections were underdeveloped, as there is much more to talk about now. I invite you to read the resubmitted manuscript to comprehensively evaluate these changes.*

*I have also included some updated text in the second paragraph of the Discussion section where I try to make the issue of habitat predictor data being (or not being) reflective of the habitat being surveyed here:

“Another important eBird data quality issue in this region is that records that are submitted to hotspots often actually represent birding effort distributed across a network of trails covering a larger area. In such cases, these checklists may contain a mixture of terra firme and floodplain data. The coordinate point used for a hotspot is therefore often not representative of the habitat in which data collection occurred, which makes using eBird data to predict species responses to the short, steep ecological gradients common in this region more challenging.*”

Lines 376-387: this paragraph is out of place in the Methods section. Either cut it or condense these ideas into a sentence or to two to preface the following ideas.

*Due to comments made by Reviewer 1, I am now testing changes in predictor importance scores statistically rather than qualitatively (this is in addition to testing the importance of the two buffer sizes as described above). I have expanded this paragraph in the resubmission and included information about the statistical tests being performed so that it now fits better in the Methods section. Please see my edits to lines 370-387 below:

“Finally, we used ‘ranger’ to calculate relative predictor importance for each model using the Gini Score [104], where relative importance value is calculated based on the mean decrease in model accuracy when the chosen predictor is excluded during the fitting process [82]. Our inclusion of predictors from both 300 m and 1 km spatial scales allowed us to directly test whether one spatial scale was preferred over another in each of the model classes. This was done by fitting a linear mixed-effects model [105] with importance score as response, predictor and the full interaction between distance and dataset as fixed effects, and species as a random effect. We also used these scores to test two predictions about relative importance shifts of habitat predictors between model classes. First, as we view the encounter rate metric to be the product of a “signal” (the occurrence process) which we care about and “noise” (the detection component) that we wish to ignore, we value models in which habitat predictors are assigned high ranks relative to detection covariates. As the acoustic dataset has more consistent levels of survey effort than the eBird dataset, we predicted that habitat predictors would be ranked more highly relative to detection covariates in the pooled models than in the eBird-only models. We tested this prediction using a linear mixed-effects model with importance as response, predictor as a fixed effect, the full interaction of “is habitat predictor” (categorical, yes or no) with dataset as a second fixed effect, and species as a random effect. Second, as we felt acoustic data provide more specific information than eBird data about where species occur within intact forest, we predicted that certain forest characteristic indicators (FCIs) should be ranked higher in importance relative to predictors measuring occurrence differences between forest and non-forest areas in the pooled model class compared to the eBird-only model class. We designated HAND and percent cover of floodplain, transition forest, and terra firme as our FCIs: HAND is the single strongest proxy of forest character in this region as flooding disturbance frequency follows an elevation gradient away from major rivers [58], and the two land cover variables are both very strongly correlated with HAND (r ≥ 0.8). As such, we feel species responses to these predictors should strongly indicate in which part of the intact forest landscape they occur. By contrast, we considered canopy height (not strongly correlated with HAND, r ~ 0.4), percent cover crops, percent cover built area, and percent cover bare ground to be more relevant to differences in species occurrence between forest and non-forest areas. We assessed this relationship using a linear mixed-effects model with importance as response, predictor as a fixed effect, the full interaction of “is FCI” (categorical, yes or no) with dataset as a second fixed effect, and species as a random effect.”*

Line 377: maybe spell out percent here.

*See preceding response for text showing this change.*

Line 379: change colon to semi-colon

*See preceding response for text showing that this line no longer contains a colon.*

Lines 399-402: This is not valid justification for your choice. Rather than acknowledging a lack of literature, explain why you selected 90 days and not shorter or longer.

*This choice has been justified using work from my colleague Carla Mere-Roncal at the same site. These lines have been edited as follows:

“We used the function “filter_repeat_visits” in the auk R package [81] to generate a subset of the eBird dataset containing between 2 and 10 repeat visits from the same observer to the same site within a period of 90 days, a period approximately equal to half the length of the two climatological seasons in the southwestern Amazon. Although we are not aware of any literature evidence documenting significant seasonal shifts in habitat utilization for our target species, this has been documented for other species of birds at Los Amigos between peak dry season and peak wet season [62], and we wished to have one closure period for each of these peak periods in addition to one each for the two transitional periods centered on November and April.”*

Lines 414-416: I’m uncertain why you made this choice. Could you perhaps provide some clarification here?

*As part of making modifications to the modeling framework as described previously, I am no longer using this select set of predictors for all species and now conduct a comprehensive model selection process for each species-model class combination. For brevity, I will avoid copying the entire description here, but in general the process involves starting with all habitat predictors (1 km summaries for the eBird models, 1 km and 300 m summaries for the other two) as possible occurrence predictors; day of year, hours of day, effort hours, protocol type (stationary or traveling), effort distance (km), and number of observers as possible detection covariates for the eBird data; and the first three of the six as possible detection covariates for the acoustic data. Strongly correlated predictors as well as those with zero variance within the dataset being assessed are removed, and then each is fit to the training data as a separate univariate model. All those that are significant in these univariate models are entered into a multivariate model to which backwards stepwise elimination is applied until WAIC stops going down, and then this is the best model.*

Lines 425-431: This information belongs in either the results or Supplemental Material.

*I have edited this text so that it now reads:*

*“We calculated the amount of anthropogenic habitat within each buffer size class as the sum of “crop” and “built area” land cover types as these are the two classes that are unambiguously the result of human influence.”

The correlation coefficient is now reported in the Results section.*

Lines 458-460: Do song rates change in the wet and dry seasons? This likely impacts detectability, no?

*Song rate changes between wet and dry seasons have been observed for other species in the Amazon, see Stouffer et al. (2013) in the manuscript. Though I am not aware of any specific research about our target species, it does seem as if most of them breed during the early wet season, so for the moment let’s assume that vocal rates are higher during that time of the year. The referenced statement is about the cumulative detection probability across all of the surveys, which is modeled by:*

$$p_{i}=1-\prod_{t=1}^{n} 1-p_{it}$$

*… where p_i_ is the i*th *site, t = 1…n is the nth visit to that site, and p_it_ is the estimated detection probability for each site-visit (Johnston et al. 2021). While seasonal changes in song rate could impact detection during the individual sampling periods (p_it_), it follows from this equation that seasonality should have a negligible impact on the cumulative detection probability at sites with a high enough level of survey effort. As a rule, the acoustic sites have substantially more effort-hours at them than the eBird sites (mean of* ~*290 effort hours per acoustic site versus ~3.6 for the eBird sites). I did go ahead and calculate detection probabilities directly for the ARU sites using the acoustic models, and for a few sites that had much less audio data available for them during one or more seasons, the cumulative detection probability did turn out to be <0.9 (though not usually by that much, e.g. ~ 0.8 or 0.7), so I now filter and remove these just like the eBird points. However, these lower detectability values were still due to differences in total site effort rather than season. In order to make this clearer to readers, I am adding the above equation and an explanation of the sampling intensity differences between data sources to this section of the manuscript as well as clarifying that the filtering step is based on cumulative detection probabilities.*

Results: Mostly well written and clear. It is comparatively sparse given the detail in the methods section. Are the results from all methods reported or at least referenced as Supplemental Material? There also seems to be a significant amount of interpretation which should be limited to the discussion.

Line 481: “respectively” is not needed.

*Removed.*

Line 492: the idea in parentheses should be explored in the Discussion and excluded here.

*Content has been moved to the Discussion*

Lines 510-520: This entire paragraph seems better suited for the Discussion.

*This paragraph has been entirely rewritten to account for the fact that I am now testing the changes in predictor values quantitatively. Here is how it reads now… I think you will agree that this is more suitable for the Results section:

“We found that 1 km summary predictors were ranked as more important on average in the eBird-only (p<0.0001) and pooled (p = 0.0001) models, while 300 m and 1 km summary predictors were ranked about equally in the audio-only models (Fig 4a). While habitat predictors were ranked more important on average than detection covariates in all model classes (Fig 4b), we found no evidence that any of the model classes was superior at assigning a higher rank to these predictors relative to the detection covariates. Our FCI habitat predictors were assigned approximately the same mean importance in all three model classes (Fig 4c). The non-forest habitat predictors were assigned a significantly higher importance in the eBird-only models compared to the audio-only models (p = 0.0062) and marginally significantly higher than the pooled models (p = 0.0719), while the pooled models assigned them approximately the same rank as the audio-only models (Fig 4c).”*

Lines 510-513: State this idea more directly; “Importance values of predictors indicates that…”

*See above.*

Line 562: Maybe spell out percent here

*This sentence no longer exists in the resubmission, but in general I have changed all instance of “%” to “percent” in this document.*

Line 571: Quantify “good job” here. This is an example of where the results section verges on the side of too much interpretation.

*The corresponding sentence in the resubmission reads:

“eBird-only models generally predicted absence…”*

Discussion: This section touches on key ideas mentioned throughout the paper, but I feel the writing and ideas could be improved and tightened. Like the Results, it feels under-developed in comparison to the Methods with assumptions made in that section unexplored and not fully acknowledged.

Lines 599-600: This sentence could be stronger and more concise; “tropical species as a whole” is clunky.

*See edited version of sentence below:*

*“Having an accessible technique for generating SDM models for Amazonian birds*

*that recapitulates known habitat associations has important implications for*

*understanding the distribution of tropical species.*

Lines 612-616: This point is not clearly communicated and I am confused by this sentence.

*This sentence has been deleted from the resubmission due to changes in the*

*methodology I used to examine changes in importance between predictor classes.*

Lines 616-618: This sentence should include a citation.

*Added citations from Table 1.*

Line 624: Is “Tropics” intended to indicate Neotropics? If so, please correct.

*No, it refers to the global Tropics, but I have clarified this.*

Lines 599-625: This entire paragraph could be tighter. I get lost in smaller details and lengthy sentences. The focus here should be on punchy, big take-aways.

*This paragraph is quite different in the resubmission, and aside from incorporating* *the new results, has also been edited for clarity. The first part, which covers a similar* *set of topics as the old paragraph, is included below:

“Our results broadly support previous work showing that small amounts of structured survey data collected over important ecological gradients can be combined with citizen science data to better model species distribution patterns [17,19,42,43]. The data pooling approach we implemented allowed pooled models to achieve higher validation scores and predictive accuracy for most species in both the encounter rate and occupancy modeling components. Inclusion of eBird data in the pooled encounter rate models, while incurring a minor predictive accuracy penalty in natural habitats relative to the acoustic-only models, allowed the models to gain insight about species responses to non-forest land cover types. They had the highest average PR-AUCs for most species, and also produced the most accurate maps (Fig 2, Fig 5). Improvement to the occupancy models was more modest from a quantitative perspective, and the pooled models were not always better than the eBird-only models for all species. Even so, the pooled models generally had better performance than the eBird-only models and were able to integrate inferences from a larger variety of spatial scales than either model class could on its own (Figs 5, 8).”*

Lines 632-638: This sentence is much too long.

*I have broken this sentence into two, as below:

“Our restriction of eBird data to checklists collected within 1 km of their recorded coordinates and (for the occupancy models) lumping clusters of eBird personal observations with nearby hotspots reduces but does not eliminate this bias. While more restrictive cutoffs would have allowed us to further reduce bias, reducing them enough to match the spatial specificity of acoustic monitors (which in tropical forest can be placed as close as 150-200m apart while remaining independent surveys [21]) would have decreased the total sample size of our eBird dataset by over half (S12 Fig), and possibly more within individual low-prevalence species.”*

Line 644: Change “This be evidence…” to “This is evidence…”

*Fixed.*

Lines 653-661: Again, this sentence is much too long.

*These lines in the original document are two sentences. However, I have broken the* *second sentence up as follows:

“We feel these results are especially relevant to other researchers in this field as the small area of coverage of our audio dataset is likely representative of that which other researchers would have access to, in Amazonia or elsewhere in the Tropics. It is far more likely that these hypothetical researchers would have access to acoustic data from a single station’s trail network, for instance, than one that covered a regional spatial extent. We believe that our study design’s targeting of the region’s most important environmental gradients is responsible for its substantial improvements in model performance despite its small spatial extent relative to the eBird dataset.”*

Line 664: Spell out TNC, also add mention of USGS Breeding Bird Survey, and other regional highly structured surveys (such as in the cited paper above).

*Fixed.*

Lines 687-689: Are any of these species known to lek, if so identify them? What about variation in vocalization rates throughout wet-dry seasons? In other parts of the neotropics, the wet season dawn choruses are characteristically reduced in length and intensity.

*See below for edits to this section, which include mentioning changes in vocal rates* *by season. I also clarify that none of our species lek, because it seems this was not* *clear in the original text:

“Our analysis preferentially looks at a small set of secretive but highly vocal species for which acoustic monitoring is an optimal survey approach, but other researchers must carefully consider factors that could limit its utility for their subject species. Careful consideration must be given to potential variation in vocal activity by season, which has been reported for some Amazonian birds [121], as well as spatial variability in vocal rates across species’ home ranges that is related to aspects of species life history [122]. For instance, while none of our species lek, vocal rates of lekking species are likely to vary dramatically within suitable habitat due to the non-random distribution of appropriate lekking sites, meaning that small variations in recorder site placement could yield dramatically different measures of site occupancy [123]. In either case, it is important to use existing domain knowledge about the subject species to assess whether the chosen survey design will yield “acoustic occupancy” rates that are comparable to true site occupancy.”*

Lines 692-695: This is interesting but not necessarily useful here.

*Some of your comments from earlier in your review suggested that I provide a*

*greater quantity of actionable information to readers who wish to employ acoustic*

*monitoring in their work on tropical birds, which I state I will do in the Introduction. In order to achieve this and make this statement more useful to readers I have rewritten it so that it gives more actionable suggestions, along with a citation to [Kovalenko, V., Doser, J. W., Bate, L. J., & Six, D. L. (2024). Paired acoustic recordings and point count surveys reveal Clark's nutcracker and whitebark pine associations across Glacier National Park. Ecology and Evolution, 14(1), e10867] in order to show a situation where this has been performed in the temperate zone:*

*“We also suggest that other researchers seeking to employ acoustic monitoring in their work on tropical bird species consider gathering a set of point-count surveys in tandem with acoustic monitoring to allow for an independent set of inferences or to directly calibrate site occupancy values, as there is recent precedent for employing this technique in temperate systems [124].”*

Lines 696-711: This paragraph introduces too much new information. Yes, it puts this study in the broader conservation context, but this can be done more concisely while also restating the major findings.

*I have slimmed down this paragraph to try to align it better with your preferences:*

*“We have demonstrated here that ARU surveys combined with machine learning acoustic classifiers can be used to rapidly collect occurrence datasets for interior forest birds; when used as part of an integrated modeling framework, this data can be used to model species habitat associations across habitats. In addition to helping researchers build a more complete picture of Amazonian diversity, such inferences will also be critical for predicting responses to anthropogenic land use change, which is a key research priority given rapid regional increases in deforestation, fire, road-building [63,128,129] and illegal artisanal gold mining along riverine corridors [64,65]. Understanding species’ current patterns of habitat utilization will be crucial for understanding potential responses to various anthropogenic pressures as well as helping to inform conservation efforts and acquisition of land for the establishment of protected areas.”*

*However, as putting this study in a broader conservation context will be important*

*for convincing people in my field to read it, I consider it a valid compromise to add*

*some of the information that was removed from this paragraph to the end of the first* *paragraph in the Methods section as part of my study area description:

“eBird survey data were collected from a much broader area within Madre de Dios, some of which is covered by intact forest but elsewhere contains open lands and areas of anthropogenic land use. Madre de Dios currently experiences significant threats from deforestation and fire, and the area bordering the Ruta Interoceánica, a newly-constructed east-west road connecting Madre de Dios and the Peruvian Andean provinces with the Brazilian state of Acre, is a major deforestation hotspot within Peru [63]. Madre de Dios has also become a hotspot for illegal artisanal gold mining in recent years; direct removal of vegetation near rivers and oxbow lakes and large inputs of mercury into the environment have yielded lasting and widespread land use change in this region, even deep into otherwise intact forest areas [15,64,65].”*
