# Supplementary material for: Combining acoustic survey and citizen science data yields enhanced species distribution models for tropical rainforest birds
Source: PLoS One. 2025 Jul 8;20(7):e0327944. doi: 10.1371/journal.pone.0327944 (PMC12237072; doi:10.1371/journal.pone.0327944)
Supplement: Attachment — Submitted filename: Response_to_Reviewers_auresp_2.docx [file pone.0327944.s025.docx]

**Manuscript PONE-D-24-56728
Response to Reviewers**

Dear Dr. Jones,

Thank you for allowing us to submit a second revised draft of the manuscript titled “Combining acoustic survey and citizen science data yields enhanced species distribution models for tropical rainforest birds” for consideration in *PLOS One*. We appreciate the time you and the two academic reviewers have dedicated to reviewing our manuscript draft and are grateful for the feedback we have received, which we have incorporated into our revised manuscript. Please see below for an itemized response to the reviewer comments for this revision. Line numbers referred to in these comments apply to those in the previous revision of the manuscript.

Many thanks,

Reid Rumelt

Ph.D. Candidate

Conservation Ecology Lab | Dr. Chris Searcy

Department of Biology | University of Miami

rumeltr@miami.edu

**Responses to Reviewer 1:**

The authors have spent considerable time and effort on their revisions and responses to questions and I find responses thoroughly addressed my original comments. I appreciate the author’s extension of the modeling to now incorporate multiple spatial summaries of the data as the importance of different buffer sizes can vary. The addition of the 1km analysis strengthens the generalizability of the study; however, it adds significant analysis and results which require minor attention.

297: GEDI lidar: GEDI should be spelled out
 *I have replaced the acronym “GEDI” with “Global Ecosystem Dynamics Investigation (GEDI)”*

392-423, Table S3, Fig4: Even though RF is extremely robust against multicollinearity, interpretation of variable importance using Gini impurity scores can be highly impacted by multicollinearity, where correlated predictors that are important can “share” importance among the highly correlated predictors resulting in importance dilution (e.g., [doi.org/10.1186/1471-2105-11-110](http://doi.org/10.1186/1471-2105-11-110)). This may make interpretability of the novel 1km predictors less reliable. Table S3 correlations are frequently >0.8 which is highly correlated and may impact variable importance, especially considering some of those variables are frequently important in fig S6. I do not think remodeling necessary, particularly because the discussion focuses more on the findings from the occupancy modeling, but the results section does include the encounter model findings and it is important to acknowledge that variable importance with the addition of the 1km covariates introduces possibly confounding effects on variable importance interpretations. If the authors chose to update the variable importances, the permutation importance option in ranger is more robust to multicollinearity than impurity.

*I think this is a very valid point to bring up. I don’t think it would be feasible to include a permutation-based assessment at this stage of the analysis as, being that this calculation is taking place within every bootstrap, doing so could lead to exponential increases to what is already a very long run time. This scared me when I was originally designing the analysis and is ultimately why I chose to use impurity scores instead, but you’re right that I need to offer some explanation for this decision. I have edited the beginning of the paragraph starting on line 392 to include the following disclaimer:

“Finally, we used the “importance” functionality within ‘ranger’ to calculate relative predictor importance within each model. We chose to measure predictor importance using the Gini impurity score implementation, which describes the mean decrease in model accuracy when the chosen predictor is excluded during the fitting process. While the impurity score calculation is highly computationally efficient, it is important to note that Gini impurity scores can suffer from importance dilution when predictors are highly correlated [105]. Our decision to use a bootstrapping procedure necessitated making this tradeoff; however, other researchers seeking to replicate these methods may be better served by using the permutation importance method, which is slower but more robust against multicollinearity [101].”

To reiterate this point, I have also added the following text at the end of the figure caption for Fig 4 :

“For computational efficiency, values represent estimated marginal means of Gini impurity scores across all model bootstraps (n=100); note that importance values may be affected by importance dilution due to multicollinearity.”*

Fig4: it would be helpful to keep the facet panel titles consistent in the figure and the caption (e.g., in C “Other” in figure but “non-forest habitat predictors” in caption). Also the case in 589-599 in the results which mirrors the caption.

*I have edited Fig4 to make the facet titles consistent with the caption and results section text. I have also changed the latter to call them “FCIs” instead of “FCI habitat predictors” to better reflect the facet text.*

Table 3: it appears the caption is below the table

*Not sure what happened to cause that, but it’s fixed now.*

**Responses to Reviewer 2:**

This is a much-improved draft, with tighter writing and nice adjustments to the modeling. I have no additional comments on the Abstract, Introduction, or Results sections, and only a few limited notes overall. The Methods section now feels clearer, and the progression of information is more intuitive. I wonder if a logical workflow diagram in the supplementary materials might help readers follow the many steps involved in data processing, pooling, and analysis. For a proof-of-concept paper like this, I think a visualization of the methods might help others who hope to implement similar tools in the tropics. This is by no means a requirement — just a suggestion if time permits. Throughout the discussion you do a really nice job of optimistically identifying the shortcomings of using eBird data from the tropics without undercutting your own work. I think part of my first-round grouchiness surrounding the assumptions of eBird data was mostly driven by inherent differences in how citizen observers collect and submit data in temperate versus tropical systems – a process which is not fully acknowledged in the literature. Since you are one of the first to use tropical eBird data for any purpose which involves rigorous models, your thoughts really set a good precedent for what others should expect or account for. This is really interesting work with clear and impactful results. It has been a pleasure to review this manuscript and watch it improve with each revision.

Line 84: Please spell out MODIS here, rather than only in the Methods section. This is my oversight — I made a similar comment regarding the reference in the Methods but didn’t catch this instance.

*No problem, I’ve edited this line so that it is now fully spelled out*

Lines 778–784: This is a fantastic point that many eBird users fail to recognize.

*Thanks… the eBird team is able to disentangle some of this lack of spatial specificity in their in-house modeling analyses because they can directly access the GPS tracks of checklists submitted through the mobile app. As much as I agree with their decision not to release this information publicly for privacy reasons, I am still envious of the cool things they can do with it.*
